# Essential structural elements in tRNA^Pro^ for EF-P-mediated alleviation of translation stalling

**DOI:** 10.1038/ncomms11657

**Published:** 2016-05-24

**Authors:** Takayuki Katoh, Ingo Wohlgemuth, Masanobu Nagano, Marina V. Rodnina, Hiroaki Suga

**Affiliations:** 1 Department of Chemistry, Graduate School of Science, The University of Tokyo, 7-3-1 Hongo, Bunkyo-ku, Tokyo 113-0033, Japan; 2 JST, PRESTO, 7-3-1 Hongo, Bunkyo-ku, Tokyo 113-0033, Japan; 3 Department of Physical Biochemistry, Max Planck Institute for Biophysical Chemistry, Goettingen 37077, Germany; 4 JST, CREST, 7-3-1 Hongo, Bunkyo-ku, Tokyo 113-0033, Japan

## Abstract

The ribosome stalls on translation of polyproline sequences due to inefficient peptide bond formation between consecutive prolines. The translation factor EF-P is able to alleviate this stalling by accelerating Pro-Pro formation. However, the mechanism by which EF-P recognizes the stalled complexes and accelerates peptide bond formation is not known. Here, we use genetic code reprogramming through a flexible *in-vitro* translation (FIT) system to investigate how mutations in tRNA^Pro^ affect EF-P function. We show that the 9-nt D-loop closed by the stable D-stem sequence in tRNA^Pro^ is a crucial recognition determinant for EF-P. Such D-arm structures are shared only among the tRNA^Pro^ isoacceptors and tRNA^fMet^ in *Escherichia coli*, and the D-arm of tRNA^fMet^ is essential for EF-P-induced acceleration of fMet–puromycin formation. Thus, the activity of EF-P is controlled by recognition elements in the tRNA D-arm.

## Abstract (web summary)

Ribosomes tend to stall during the translation of consecutive proline residues, which can be rescued by the co-translational factor EF-P. Here the authors identify a structural element of tRNA^Pro^ responsible for specific recognition by EF-P and stimulation of Pro-Pro peptide bond formation.

## Graphical abstract

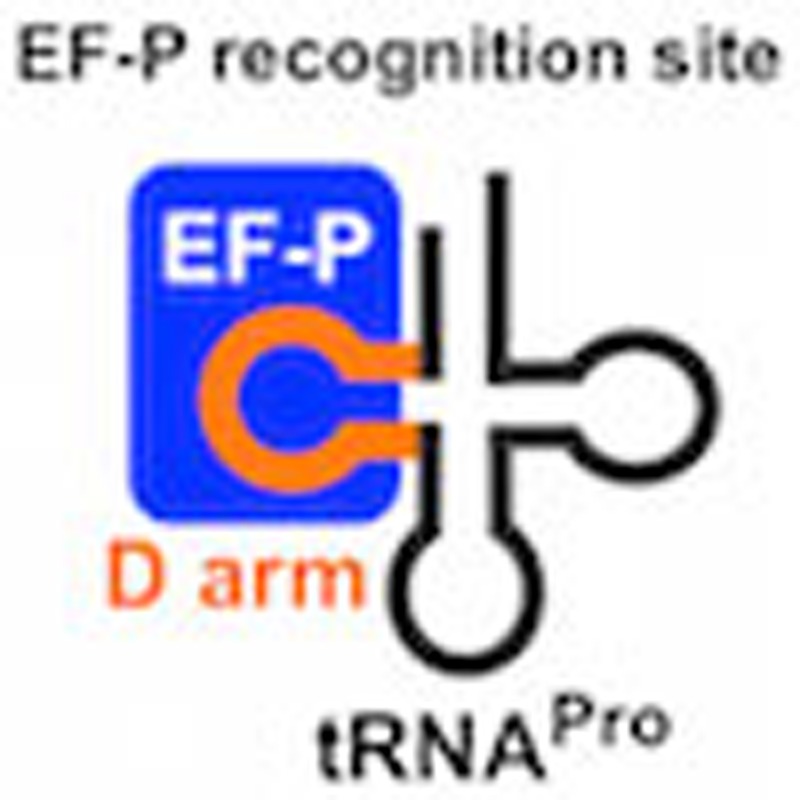

The EF-P bacterial translation factor was discovered in the 1970s and was reported to stimulate peptide bond formation between initiator fMet–tRNA^fMet^ and puromycin (Pmn), an analogue of the 3′-end of aminoacyl-transfer RNA^1^,^2^. Archaeal and eukaryotic EF-P homologues (aIF5A and eIF5A, respectively) were subsequently found^3^, suggesting that the EF-P-family proteins are evolutionarily conserved^4^,^5^,^6^,^7^,^8^. EF-P homologues in many organisms are posttranslationally modified. In EF-P from *E. coli*, Lys34 is modified to lysyl-hydroxylysine by three enzymes, EpmA, EpmB and EpmC (formerly known as YjeK, YjeA and YfcM, respectively)^5^,^6^,^8^,^9^,^10^. Lysylation is required for the full activity of EF-P^11^,^12^. The knockout of genes coding for EF-P, eIF5A or their modifying enzymes affects a variety of biological functions or even causes lethality^13^,^14^. On the other hand, it is also known that *E. coli* reconstituted translation systems that lack EF-P, such as the PURE system^15^, can be used to express many different proteins, suggesting that EF-P is not essential for protein synthesis. This apparent paradox was resolved when two groups simultaneously demonstrated that EF-P is a specialized factor required for translation of an messenger RNA subset containing polyproline motifs^11^,^12^. When the ribosome encounters such motifs, it tends to stall, because peptide bond formation between three or more Pro residues, or two Pro residues in certain contexts, is very slow^11^,^12^,^16^,^17^,^18^,^19^. EF-P rescues the stalled ribosomes to yield full-length proteins^11^,^12^. In *E. coli*, ∼270 proteins out of ∼4,000 proteins contain PPG or PPP motifs and their synthesis may be affected by the lack of functional EF-P^12^, explaining the pleiotropic or lethal phenotypes of deletion strains lacking EF-P/eIF5A or their modifying enzymes.

The structure of *Thermus thermophilus* 70S ribosomes in complex with EF-P and tRNA^fMet^ revealed that EF-P binds between the exit (E) and the peptidyl (P) sites of the ribosome and interacts with the phosphate backbone of tRNA^fMet^ at the acceptor stem, D-arm and the anticodon stem (Fig. 1a,b). Although these interactions may stabilize tRNA^fMet^ in the P site^20^, the mechanism for the acceleration of peptide bond formation between fMet–tRNA^fMet^ and Pmn remained unclear. Moreover, the structure of the ribosome in complex with EF-P and its cellular substrate Pro-tRNA^Pro^ is currently not available, and the potential contribution of interactions between EF-P and the mRNA codon in the E site is uncertain^20^,^21^. Thus, it is not known whether interactions between EF-P and peptidyl-Pro-tRNA^Pro^ accelerate Pro-Pro formation. Linear free energy relationships of peptide bond formation with a variety of Pro analogues on the ribosome and in solution suggest that the major determinant of the impaired reaction between consecutive Pro residues is the unfavourable steric arrangement of peptidyl-Pro-tRNA^Pro^ in the P site^22^. The peptidyl moiety attached to the P-site tRNA^Pro^ mediates ribosome stalling^12^,^16^,^17^,^19^. EF-P appears to act by entropic steering of Pro-tRNA towards a catalytically productive orientation in the peptidyl transferase centre of the ribosome^6^. However, how this is achieved remains unknown. This prompted us to address the following open questions: How important is tRNA^Pro^ to elicit the stimulatory effect of EF-P on peptide bond formation? Does the codon–anticodon interaction of tRNA^Pro^ contribute? Is the peptide bond formation with other secondary amino acids, such as *N*-methyl-amino acids, enhanced by EF-P? If the sequence of tRNA^Pro^ is important, which parts of tRNA^Pro^ are recognized by EF-P?

To identify the elements of peptidyl-Pro-tRNA^Pro^ steering the interactions with EF-P in the ribosome complex, we used a reconstituted *in-vitro* translation system coupled with flexizyme technology, referred to as the FIT (flexible *in-vitro* translation) system^23^. We analysed the EF-P effect for various tRNA mutants engineered to decode sequential Pro codons. Furthermore, time-resolved experiments by quench flow techniques attributed the global translation effects to the step of peptide bond formation. We conclude that the function of EF-P critically depends on two constraints: (1) the P and A sites of the peptidyl transferase centre must be occupied by a peptidyl-Pro-tRNA and a sufficiently unreactive substrate carrying Pro or other secondary amino acid, respectively; and (2) the P-site tRNA must bear the tRNA^Pro^ D-loop closed by a stable stem (for example, tRNA^Pro^ D-arm sequence) regardless of the sequence of the remaining part of the tRNA molecule; among *E. coli* tRNAs, only tRNA^Pro^ and tRNA^fMet^ share such sequences. Thus, the action of EF-P is promoted by the presence of a Pro and the recognition elements in the P-site tRNA, which explains the specificity of the factor for polyproline motifs.

## Results

### EF-P recognizes all isoacceptors of Pro-tRNA^Pro^

First, the translation enhancement by EF-P was reproduced in the FIT system using three different mRNA constructs (mR1, mR2 and mR3) coding for Pro-Gly-Gly, Pro-Pro-Gly and Pro-Pro-Pro, respectively (Supplementary Fig. 1). With such short sequences, the proline-induced stalling results in a significant peptidyl-tRNA drop-off, which is proportional to the duration of stalling. EF-P addition reduces stalling and thus increases the final product level, which enabled us to assess the EF-P effect through quantifying the peptide yield of translation. Pro insertion into the respective peptides (P1, P2 and P3) depends on exogenous Pro-tRNA^Pro1^ prepared by flexizyme technology^23^,^24^. Translation was carried out by adding the Pro-tRNA^Pro1^ and the DNA template for the corresponding mRNA to the FIT system^23^ containing ribosomes, translation factors, native tRNA mixture, five aminoacyl-tRNA synthetases (ARSs; MetRS, LysRS, GlyRS, TyrRS and AspRS) and five amino acids (Met, Lys, Gly, Tyr and [^14^C]Asp) in the presence or absence of EF-P. In this translation assay optimized with respect to incubation time and EF-P concentration (Supplementary Fig. 2), EF-P significantly improved the translation yields of peptides with consecutive Pro residues (3.5- and 14.4-fold increase for P2 and P3, respectively), whereas the yield of the peptide P1 with a single Pro was not affected, consistent with previous studies^11^,^12^,^16^. To investigate the impact of tRNA structural features on the function of EF-P, we prepared eight tRNA transcripts with the sequences of *E. coli* tRNA^Ser4^, tRNA^Trp^, tRNA^Leu2^, tRNA^Ile1^, tRNA^Ala2^, tRNA^Pro1^, tRNA^Pro2^ and tRNA^Pro3^, and charged them with Pro by means of flexizyme technology. The corresponding mRNA templates, referred to as mR2-XXX_2_, each contained two tandem XXX codons that were decoded by the respective Pro-tRNA bearing the complementary anticodon (Fig. 2a). EF-P improved translation yield only when tRNA^Pro^ isoacceptors (tRNA^Pro1^, tRNA^Pro2^ and tRNA^Pro3^) were used to decode the tandem Pro codons, whereas no enhancement was observed with other tRNAs (Fig. 2b and Supplementary Fig. 3a). This result shows that the EF-P alleviates translation stalling with tRNA^Pro^ isoacceptors only.

To determine whether the tRNA^Pro^ codon–anticodon interaction is responsible for the enhancement, we prepared chimeric tRNAs derived from the sequences of tRNA^Pro1^ or tRNA^Ser4^, but with the anticodons swapped (CGG or GGA) (Fig. 2c and Supplementary Fig. 3b). We then tested the effect of EF-P on translation of mR2-CCG_2_ or mR2-UCC_2_. EF-P was able to enhance translation regardless of the anticodon, but only if Pro was charged onto an isoacceptor of tRNA^Pro1^; EF-P did not improve the yield of translation with Pro-tRNA^Ser4^_CGG_ or Pro-tRNA^Ser4^_GGA_ (Fig. 2c and Supplementary Fig. 3b). This result suggests that codon–anticodon interactions do not serve as determinants for EF-P function, but rather some other part of the tRNA structure is crucial.

### Requirement for Pro and tRNA^Pro^

It is not known what makes the ribosome stall during the synthesis of poly-Pro sequences. As Pro is the only secondary proteinogenic amino acid, one could hypothesize that this structural feature serves as a specific recognition element for EF-P. If so, then EF-P may also enhance peptide bond formation with other secondary amino acids such as *N*-methyl-amino acids. To test this notion, we chose two non-proteinogenic secondary amino acids, L-*N*-methylalanine (^Me^Ala) and L-*N*-methylthreonine (^Me^Thr), to study their tandem incorporation in the presence and absence of EF-P using mR2 (Fig. 3a,b and Supplementary Fig. 3c); as a positive control, we also examined consecutive insertion of Pro into the peptide. The unnatural amino acids were charged onto tRNA^Pro1^, which decoded two sequential CCG codons in the mRNA. To our surprise, EF-P had almost no effect when two identical *N*-methyl-amino acids were inserted, compared with the Pro-Pro control (Fig. 3b). This result indicates that EF-P selectively enhances Pro incorporation.

We then investigated which of the two Pro residues, the first or the second, is needed to elicit EF-P activity. We designed four templates (mR2 and mR4-6) which encode Pro-Pro (CCG-CCG), Pro-^Me^Thr (CCG-UCC), ^Me^Thr-Pro (UCC-CCG) or ^Me^Thr-^Me^Thr (UCC-UCC) (Fig. 3a, mR2, mR4, mR5 and mR6, respectively). We note that EF-P enhances translation when Pro is encoded by a UCC codon read by tRNA^Pro1^_GGA_ (Fig. 2c); hence, tRNA^Pro1^_GGA_ should be functionally identical to tRNA^Pro1^_CGG_. In this system, EF-P stimulated the synthesis of Pro-Pro and Pro-^Me^Thr but not of ^Me^Thr-Pro and ^Me^Thr-^Me^Thr (Fig. 3c and Supplementary Fig. 3d). Interestingly, EF-P enhanced Pro-^Me^Thr insertion by fourfold (P4), which is more than the effect measured for Pro-Pro (P2) in this peptide sequence context.

To further test which part of the P-site tRNA^Pro^ molecule is essential for the enhancement by EF-P, we designed a combinatorial permutation experiment where the body sequences of Pro-tRNA^Pro1^_CGG_ and ^Me^Thr-tRNA^Pro1^_GGA_ were changed to those of tRNA^Ser4^_CGG_ and tRNA^Ser4^_GGA_, respectively (Fig. 3d and Supplementary Fig. 3e). EF-P enhanced Pro-^Me^Thr synthesis when Pro-tRNA^Pro1^_CGG_ was present in the P site regardless of the tRNA in the A site, although ^Me^Thr-tRNA^Pro1^_GGA_ gave higher enhancement than ^Me^Thr-tRNA^Ser4^_GGA_ in the A site. On the other hand, EF-P was unable to facilitate Pro-^Me^Thr synthesis when Pro-tRNA^Ser4^_GGA_ was present in the P site regardless of the tRNA body structure or the secondary amino acid in the A site (Fig. 3d and Supplementary Fig. 3e). These results indicate that EF-P recognizes both the peptidyl-Pro moiety and the tRNA^Pro^ body sequence in the P site to accelerate the slow elongation caused by Pro-Pro or Pro-^Me^Thr elongation^25^.

### EF-P recognition elements are in the D-arm of tRNA^Pro^

In the X-ray crystal structure of the ribosome·EF-P·fMet–tRNA complex, EF-P contacts the backbone of the D-arm, acceptor and anticodon stem regions of tRNA^fMet^. As we showed above, the action of EF-P depends on the presence of tRNA^Pro^ in the P site. To determine which part of tRNA^Pro^ is essential, we designed six chimeric tRNA constructs (tRNA^chim^) based on the tRNA^Pro1^ sequence, where the D-arm, acceptor stem or anticodon stem regions were independently substituted with those of tRNA^Ser4^ or tRNA^Leu2^ (Fig. 4a). Pro was then charged onto the respective tRNA^chim^ by the flexizyme and used to decode tandem CCG codons. EF-P did not enhance peptide synthesis when the D-arm of tRNA^Pro1^ was substituted with that of tRNA^Ser4^ or tRNA^Leu2^ (tRNA^chim^ 1 or 2) (Fig. 4b and Supplementary Fig. 3f). In contrast, the effect of the substitutions of acceptor stem or anticodon stem was much less pronounced (tRNA^chim^ 3–6). We note that the translation yield in the absence of EF-P was similar for all mutant and WT tRNAs, indicating that mutant tRNAs were functionally active. These results suggest that the D-arm of tRNA^Pro^ contains the major determinants for EF-P recognition.

To further investigate the functional importance of the D-arm bases, we first prepared eight D-arm single-point mutants (C13G, G15A, C16U, C17aU, U17bG, G18U, U20A and A21G; Fig. 4c and Supplementary Fig. 3g), four double-point mutants (C13G/C16U, C13G/U20A, C16U/C17aU and C17aU/U20A) and three triple-point mutants (C13G/C16U/C17aU, C13G/C17aU/U20A and C16U/C17aU/U20A; Supplementary Fig. 4a). These tRNA mutants were charged with Pro and used to synthesize the P2 peptide. Among the single-point mutants, only the C13G mutation at the edge of the D-stem significantly decreased the stimulation by EF-P, whereas other mutations in the D-loop had no effect (Fig. 4c and Supplementary Fig. 3g). Similarly, the double- and triple-point mutants containing a mutation at position 13 decreased the sensitivity to EF-P, whereas all others were similar to the wild-type tRNA^Pro^ (Supplementary Fig. 4a). The effect of the C13G mutation was robust under all conditions tested (Supplementary Fig. 2a,b) and specific for EF-P-induced translation (Supplementary Fig. 3g). These results indicate that among the D-loop bases the C13/G22 base pair plays a critical role in mediating the enhancement by EF-P. We further validated the importance of the D-stem in the interaction with EF-P on translation of the amino-terminal part of the flagellar protein FLK, which contains PPP and PPG sequences (Supplementary Fig. 5). EF-P significantly improved the FLK yield, whereas only slight improvement was observed for the C13G mutants. This result provides further support for the conclusion that the D-arm structure in tRNA^Pro^ is crucial for EF-P activity.

We next constructed tRNA^Pro1^ variants focusing on the mutations at positions 13/22 and 12/23 (Fig. 4a), and again measured the affect of EF-P on P2 synthesis. With tRNA variants that maintained the stable base pair (G13/C22 and C12/G23), translation enhancement by EF-P was stronger than with those containing weakened (U13/A22, A13/U22, U12/A23 and A12/U23) or mismatched base pairs (Fig. 4d,e and Supplementary Fig. 3h,i). We also tested the impact of the D-loop size using two additional tRNA^Pro1^ variants, with a 1-nt shorter (ΔC17a) or 1-nt longer (+17cU) D-loop, respectively (Fig. 4f). These loop size alterations essentially abolished the EF-P-mediated enhancement of translation (Fig. 4g and Supplementary Fig. 3j).

Together, these data indicate that the size of the D-loop in combination with the stable D-stem probably serve as critical EF-P recognition elements in the P site-bound peptidyl-Pro-tRNA^Pro^. The three naturally occurring isoacceptors of tRNA^Pro^ have a 9-nt D-loop and a stable 4-bp D-stem with two G/C pairs at positions of 13/22 and 12/23. Among the 46 *E.coli* tRNAs, this structure is shared by only the three Pro isoacceptors and tRNA^fMet^, and would be a key factor for selective enhancement of translation by EF-P (Fig. 4h and Supplementary Table 1). Robust EF-P activity was observed with quite a number of mutations in the D-loop, provided the length of the loop was maintained (Fig. 4c and Supplementary Figs 3g and 4a). In addition, different base pairs in the D-stem were tolerated as long as the stability of the D-stem was maintained (Fig. 4d,e and Supplementary Fig. 3h,i). Even though a posttranscriptional dihydrouridine modification occurs at position 20 in the natural *E. coli* tRNA^Pro^ (Supplementary Fig. 4b), the activity of an *in-vitro* transcript lacking this modification is very similar to that of the fully modified tRNA (Supplementary Fig. 4c), including EF-P recruitment and catalysis^22^, suggesting that the dihydrouridine modification is not involved in EF-P recognition. These results allow us to hypothesize that EF-P does not recognize the D-loop of tRNA^Pro^ in a sequence-specific manner, but rather senses the loop size closed by the stable D-stem via phosphate backbone interactions.

### EF-P recruitment by substituting D-arm of inactive tRNAs

To further verify the recognition determinants in the D-loop in the absence of possible interactions with other parts of tRNA^Pro^, we prepared chimeric tRNAs derived from tRNA^Ser4^ and tRNA^Ala2^, which are normally not recognized by EF-P. The parental wild-type tRNA^Ser4^ has an 11-nt D-loop and a 3-bp D-stem, whereas the tRNA^Ala2^ has an 8-nt D-loop and a 4-bp D-stem. We replaced the native D-arm and anticodon sequences of these tRNAs with the D-arm of tRNA^Pro1^ and the CGG anticodon capable of decoding the CCG Pro codon (Fig. 5a,c). These chimeric tRNAs were charged with Pro and used for the translation of the mR2 mRNA.

Although translation was insensitive to EF-P when the single compensatory mutation of C12G/G23C in tRNA^Ser4^ (tRNA^Ser4^-1) was used, stabilization of the D-stem by the G13C/A22G mutation (tRNA^Ser4^-2), which changed the loop size to 9 nt, conveyed a small EF-P-dependent activation effect even though the D-loop sequence was quite different (Fig. 5b and Supplementary Fig. 3k). The EF-P-mediated translation enhancement was fully restored when the intact stable D-stem and D-loop derived from tRNA^Pro1^ were introduced into tRNA^Ser4^ (tRNA^Ser4^-3). A similar trend was also seen in the chimeric tRNA^Ala2^ (tRNA^Ala2^-3), in which the stable 4-bp D-stem and the 9-nt D-loop were engineered by insertion of a single base (17aU) even though the D-loop sequence was not identical to that of tRNA^Pro^ (Fig. 5d and Supplementary Fig. 3l).

We further tested Pro incorporation using tRNA^Val2A^ that comprised the same length of D-loop and D-stem as tRNA^Pro^ (a 9-nt D-loop and a 4-bp D-stem) (Fig. 5e,f and Supplementary Fig. 3m). EF-P slightly accelerated Pro incorporation with the WT tRNA^Val2A^, but the effect was smaller than that with tRNA^Pro1^ due to the weaker base pair (U/A) at the 12/23 than that of tRNA^Pro1^ (G/C). Given that U12/A23 and U13/A22 mutants of tRNA^Pro1^ also showed slight enhancement (Fig. 4d,e), U/A base pairs at these positions weakly contribute to EF-P function. The EF-P effect was fully restored by stabilization of the D-stem by the U12G/A22C mutation (tRNA^Val2A^-1), whereas the effect was abolished by destabilization of the D-stem by U12A mutation (tRNA^Val2A^-2).

These results suggested that alterations of the D-loop sequence can be tolerated, but the full EF-P effect can be only achieved when the geometry of the tRNA^Pro^ D-loop is maintained, which presumably allows for EF-P recruitment. These results further support the conclusion that the major recognition determinant in tRNA^Pro^ comprises the D-loop closed by the stable D-stem.

### EF-P recognizes the D-arm structure of tRNA^fMet^

As discussed above, tRNA^fMet^ and the three tRNA^Pro^ isoacceptors share the same D-arm motif, that is, a 9-nt D-loop closed by a stable 4-bp D-stem with two G/C pairs at 12/23 and 13/22 (Fig. 4h and Supplementary Table 1). As EF-P accelerates not only the synthesis of poly-Pro motifs but also the fMet–Pmn reaction^1^,^12^, it is likely to be that EF-P also recognizes the D-loop of fMet-tRNA^fMet^. To test this hypothesis, we first studied Pro incorporation using tRNA^fMet^_CGG_ derivatives (tRNA^fMet^_CGG_-1-4) containing D-arm substitutions along with the CGG anticodon (Fig. 6a) and charged with Pro. As expected, EF-P enhanced translation of P2 peptide when Pro-tRNA^fMet^_CGG_ was used in the assay (Fig. 6b and Supplementary Fig. 3n, tRNA^fMet^_CGG_-1 versus tRNA^Pro1^_CGG_). Substitution of the D-arm of tRNA^fMet^ with those of tRNA^Ser4^ and tRNA^Leu2^ or introduction of the C13G or ΔC17a mutations (Fig. 6b and Supplementary Fig. 3n, 2–5) eliminated EF-P-mediated activation. These results indicated that Pro-tRNA^fMet^_CGG_, similar to Pro-tRNA^Pro1^_CGG_, is able to recruit EF-P.

The above result of the translation experiment using Pro-tRNA^fMet^_CGG_ prompted us to test fMet–Pmn formation using fMet-tRNA^fMet^_CAU_ or its mutants. We used four tRNA^fMet^_CAU_ mutants in addition to the wild-type tRNA^fMet^_CAU_ (Fig. 6c, 1–4). The initiation complex of ribosome containing f[^35^S]Met-tRNA^fMet^_CAU_ or its mutants was incubated with Pmn in the presence or absence of EF-P (Fig. 6d and Supplementary Fig. 3o, WT and 1–4). EF-P enhanced the formation of fMet–Pmn dipeptide over background when the wild-type fMet-tRNA^fMet^_CAU_ was used, although its enhancement by 1.8-fold was less than the 4-fold effect observed in P2 translation. The Pmn reaction with tRNA^fMet^_CAU_ mutants was insensitive to EF-P (Fig. 6d and Supplementary Fig. 3o).

### Kinetic effect of the D-arm mutants

Although the data described above were generated by the analysis of translation yields of 15-mer peptide containing consecutive Pro residues, they are expected to represent the rate-limiting step of translation, which is most likely to be the formation of the ProPro-Gly peptide bonds. We further tested the effect of the D-arm mutations by measuring the rate of Pro-Gly formation between fMet-Pro-tRNA and Gly-tRNA, which is known to be accelerated by EF-P^12^,^22^. We tested four representative D-arm variants derived from tRNA^Pro1^_CGG_ (Fig. 7a) and tRNA^Ser4^_CGG_ (Fig. 7b), and charged the tRNA constructs with Pro. The wild-type or mutant Pro-tRNA was incubated with EF-Tu·GTP and added to the ribosome initiation complex programmed with an mRNA coding for fMet-Pro-Gly tripeptide and containing f[^14^C]Met-tRNA^fMet^ in the P site, which resulted in the formation of fMet-Pro-tRNA^Pro^. Next, EF-G was added to form posttranslocation complexes that were subsequently purified by size-exclusion chromatography. Posttranslocation complexes (fMet-Pro) were rapidly mixed with [^3^H]Gly-tRNA^Gly3^·EF-Tu·GTP to measure the rate of Pro-Gly formation using a quench-flow technique. The rate constant (*k*_obs_) was determined by exponential fitting of time courses (Supplementary Fig. 6a–f).

With the wild type and all tRNA^Pro^ mutants, the time courses of fMet-Pro-Gly formation in the absence of EF-P were similar, with a *k*_obs_ in a range of 1–2 s^−1^ (Supplementary Fig. 6a–f). This indicates that the variations of the D-arm sequence did not affect the intrinsically slow Pro-Gly formation when EF-P is absent. In the presence of EF-P, the rates of peptide bond formation with the native tRNA^Pro1^_CGG_ (WT) and the tRNA^Ser4^_CGG_ variant (4) bearing the D-arm derived from tRNA^Pro1^ increased (Fig. 7c,d) by about 18- and 8-fold, respectively. In contrast, the reactions with tRNA^Pro1^_CGG_ mutants and native tRNA^Ser4^_CGG_ were hardly affected by EF-P addition (Fig. 7c, 1–3 and Fig. 7d, WT, respectively). As expected, the kinetically resolved EF-P effects are much larger than those obtained from the translation yield analysis. These results fully support the hypothesis that EF-P recognizes the D-arm structure of the peptidyl-Pro-tRNA to enhance the rate of Pro-Gly formation. Thus, we conclude that the EF-P enhancement observed in the translation of the 15-mer peptide should faithfully reflect the identity elements on tRNA required for the productive recruitment of EF-P.

## Discussion

Our experiments using Pro-tRNA derivatives identify the structural elements in tRNA^Pro^ that are required for the EF-P function: the D-loop structure closed by the stable base pairs in the D-stem of peptidyl-Pro-tRNA^Pro^ (Fig. 4a–e). Many point mutations in the D-loop are tolerated (Fig. 4c and Supplementary Figs 3g and 4a) but alteration of the D-loop size inhibits EF-P function (Fig. 4f). These observations suggest that the overall loop structure of the D-loop is the essential recognition element for EF-P. In contrast, alterations in the acceptor stem, anticodon stem and anticodon of tRNA^Pro^ have only modest effects. In fact, the chimeric derivatives of tRNA^Ser^, tRNA^Leu^ (Figs 4a,b and 5a,b, and Supplementary Fig. 3f,k), tRNA^Ala^ (Fig. 5c,d and Supplementary Fig. 3l) and tRNA^Val2A^ (Fig. 5e,f and Supplementary Fig. 3m) in which the D-arm sequence of tRNA^Pro^ is implanted have a gain-of-function phenotype.

The structure of Pro in the P-site peptidyl-Pro-tRNA^Pro^ also plays a critical role in EF-P function, that is, peptide bond formation with other secondary amino acids such as *N*-methyl-amino acids is not enhanced by EF-P (Fig. 3 and Supplementary Fig. 3c). Importantly, the kinetic measurement of EF-P-stimulated Pro-Gly formation using various ‘active' and ‘inactive' derivatives of Pro-tRNA^Pro^_CGG_ and Pro-tRNA^Ser^_CGG_ supports the results observed for 15-mer peptide expression (Fig. 7). This detailed analysis also showed that all tested tRNA constructs formed stable posttranslocation complexes and showed similar rates of peptide bond formation in the absence of EF-P, suggesting that the alterations in these tRNAs did not affect their activity in translation. These results also validate the loss-of-function experiments in which the absence of an EF-P effect may have otherwise been explained by a strongly impaired translation reaction.

EF-P was originally identified as a protein factor that stimulates peptide bond formation between fMet and Pmn, and the D-arm of tRNA^fMet^ is virtually identical to that of tRNA^Pro^ (Fig. 4h); consistent with this observation, the effect of EF-P on translation in the presence of Pro-tRNA^fMet^_CGG_ is dependent on the presence of the correct D-arm (Fig. 6a,b and Supplementary Fig. 3n). Furthermore, this was also the case for fMet–Pmn formation with fMet-tRNA^fMet^_CAU_, although the observed degree of fMet–Pmn acceleration is less than that for translation. In addition, for the fMet–Pmn reaction, tRNA^fMet^ variants with the D-loop sequences derived from tRNA^Ser4^ and tRNA^Leu2^ do not support EF-P function (Fig. 6c,d). Based on these results we propose that EF-P recognizes the shape of the phosphate backbone of the D-loop in the P-site peptidyl-Pro-tRNA^Pro^, to accelerate the formation of Pro-Xaa peptide bonds, in which Xaa is a poor A-site substrate such as Gly, Pro or other secondary amino acids. Comparison of the D-arm sequence of *E. coli* tRNA^Pro^ with that of other prokaryotic species shows that the 9-nt loop and stable 4-bp stem structure is well conserved among them (Supplementary Table 2). Although U12/A23 and U13/G22 pairs are found in several species, these base pairs would also be tolerated considering that the *E. coli* tRNA^Pro1^ mutant and tRNA^Val2A^ with these sequences elicited a modest EF-P effect (Fig. 4d,e and Fig. 5e,f). Therefore, D-arm recognition by EF-P may be similar in diverse prokaryotic organisms. Eukaryotic translation factor eIF5A is also able to stimulate incorporation of consecutive Pro amino acids^26^ and peptide bond formation between Met-tRNA_i_^Met^ and Pmn^27^,^28^,^29^. Although the D-stem of eukaryotic tRNA^Pro^ is generally shorter by 1 bp than in the prokaryotic counterparts, a 9-nt D-loop closed by a 3-bp stem is well conserved among eukaryotic species (Supplementary Table 2). Thus, it is likely to be that eIF5A recognizes the phosphate backbone of the D-loop in peptidyl-Pro-tRNA^Pro^ in a similar way as EF-P. Hydroxyl radical probing data of the P-site tRNA region proximal to eIF5A broadly suggested potential contacts of eIF5A with the acceptor stem, D-loop, anticodon stem and T-loop of the P-site-bound tRNA in 80S ribosome^26^,^30^. However, such analyses did not reveal the critical contacts of the D-arm essential for the EF-P enhancement, probably because the ground-state mapping of the EF-P-tRNA contacts might not reflect the kinetic importance of the D-arm contributing to the EF-P enhancement. Therefore, appropriate biochemical studies similar to the experiments reported in this work would be necessary to confirm the importance of the D-arm in eukaryotes.

On the other hand, the length of the D-loop and D-stem of archaeal tRNA^Pro^ differs among species (Supplementary Table 2), suggesting that aIF5A, the archaeal counterpart of EF-P, may not recognize the D-arm of tRNA^Pro^ in the same manner. If so, archaeal aIF5A might be evolutionarily distant from prokaryotic EF-P and eukaryotic eIF5A in terms of its tRNA recognition mechanism; this question should be addressed in future experiments using the flexizyme technology and archaeal translation components. Notably, it was reported that a prokaryotic translation factor, EttA, binds to the E site of the ribosome and bridges the ribosomal L1 stalk and the P-site tRNA^31^, involving the EttA recognition of C17a and U17b in the D-arm of P-site tRNA^fMet^. Although these bases are also shared by tRNA^Pro1^ and tRNA^Pro2^, point mutations at these positions in tRNA^Pro1^ did not affect the function of EF-P (Fig. 4c), indicating that the binding mode of EF-P is different from that of EttA. Apart from EF-P and EttA, there are a growing number of translation factors such as RbbA and HflX that bind to the E site^32^,^33^. The cellular functions of these factors are different: EttA senses the energetic status of the cell and gates the entry into the elongation cycle of translation, HflX splits ribosomes under stress and RbbA ejects the E-site tRNA from the ribosome. The use of recognition motifs such as presented here for EF-P might be a common motif to orchestrate these diverse functions.

There is as yet an open question as to what is the mechanism of EF-P acceleration of Pro-Pro bond formation. A plausible hypothesis is that via the interaction of EF-P with the D-arm of tRNA^Pro^ in the P site, the ester's carbonyl group Pro-tRNA^Pro^ is appropriately positioned in the peptidyl transfer centre and this facilitates the nucleophilic attack of the secondary amino group on Pro or an *N*-methyl-amino acid, which are intrinsically poorer nucleophiles compared with the primary amino groups of other proteinogenic amino acids. Although this hypothesis does not tell us the mechanism at the molecular level, structural studies with a higher resolution may reveal this in more detail.

## Methods

### Preparation of aminoacyl-tRNAs

All tRNA constructs used for Pro and *N*-methyl-amino acid incorporation were prepared by *in vitro* transcription, except for the experiment in Supplementary Fig. 4c. Template DNAs for T7 RNA polymerase transcription were prepared using *Taq* DNA polymerase by extension of forward and reverse primers pairs (Supplementary Table 3), followed by PCR using forward and reverse PCR primers (Supplementary Table 3). The DNA products were purified by phenol extraction and ethanol precipitation, and then transcribed at 37 °C for 12 h in a 200-μl reaction mixture consisting of 40 mM Tris-HCl (pH 8.0), 22.5 mM MgCl_2_, 1 mM dithiothreitol (DTT), 1 mM spermidine, 0.01% Triton X-100, 0.04 U μl^−1^ RNasin RNase inhibitor (Promega), 3.75 mM NTP mix and 5 mM CMP or GMP, or AMP, depending on the 5′-end nucleotide of the tRNA. The resulting RNAs were treated with RQ1 DNase (Promega) for 30 min at 37 °C and then purified by 8% denaturing PAGE containing 6 M urea.

Aminoacylation of the tRNAs was carried out on ice in reaction mixtures containing 50 mM HEPES-KOH (pH 7.5), 600 mM MgCl_2_, 20% dimethyl sulfoxide, 25 μM dFx, 25 μM tRNA and 5 mM proline dinitrobenzyl ester (DBE), *N*-methylalanine DBE or *N*-methylthreonine DBE. Reactions with *N*-methylalanine and *N*-methylthreonine were performed for 6 h, those with proline for 2 h. Aminoacyl-tRNA was precipitated by ethanol and dissolved in 1 mM sodium acetate (pH 5.2) to a concentration of 250 μM.

The [^14^C]Pro-labelled native tRNA^Pro1/Pro2/Pro3^ mixture used in Supplementary Fig. 4c was prepared as follows. Total tRNA was purchased from Roche. For aminoacylation, 400 μM total tRNA, 5% (v/v) S100 fraction as source of ARSs, 3 mM ATP and 25 μM [^14^C]Pro were incubated for 45 min in aminoacylation buffer (50 mM HEPES-KOH (pH 7.8), 70 mM NH_4_Cl, 30 mM KCl and 20 mM MgCl_2_). [^14^C]Pro-tRNA^Pro^ was phenolized and precipitated with ethanol. Aminoacyl-tRNA was dissolved in water and added to EF-Tu·GTP to form ternary complex [^14^C]Pro-tRNA^Pro^·EF-Tu·GTP and separated from uncharged total tRNA by size-exclusion chromatography on two tandem Superdex 75 columns (GE Healthcare). Isolated ternary complexes were phenolized and [^14^C]Pro-tRNA^Pro^ was precipitated with ethanol.

### Preparation and analysis of lysylated EF-P

*E. coli EF-P* gene was cloned into a modified pET28a vector that has PreScission protease cleavage site instead of thrombin cleavage cite. *E. coli EpmA* and *EpmB* genes were cloned into pETDuet vector. These proteins were expressed in *E. coli* Rosetta2 (DE3) cells with 0.5 mM isopropyl-β-D-thiogalactoside, followed by cell lysis by sonication and loading onto a Ni-NTA column to purify the histidine-tagged EF-P. The column was washed with Buffer A containing 20 mM Tris-HCl (pH 8.0), 200 mM NaCl, 2 mM imidazole and 1 mM 2-mercaptoethanol, and then the protein was eluted by Buffer A with 300 mM imidazole. The eluate was dialysed against the Buffer A and the histidine tag of EF-P was cleaved using Turbo3C protease at 4 °C overnight. The protein was reloaded on a Ni-NTA column and the flowthrough and wash fractions were pooled. Finally, the protein was further purified on a Resource Q column (GE Healthcare).

Lysylation of EF-P was confirmed by mass spectrometric analysis according to the following procedure (Supplementary Fig. 7). Fifty to 100 pmol EF-P was separated on a 4–20% gradient SDS–PAGE gel, stained with Coomassie Blue and excised for in-gel proteolysis. Cysteines were reduced with 10 mM DTT for 30 min at 56 °C and alkylated with 55 mM iodoacetamide for 60 min at room temperature in the dark. EF-P was proteolysed with 12.5 ng μl^−1^ trypsin for 16 h at 37 °C. Extracted peptides were dried and dissolved in 5% acetonitrile/0.1% formic acid. Next, proteolyszed EF-P was analysed by reverse-phase HPLC–electrospray ionization–tandem mass spectrometry using a Dionex Ultimate 3000 HPLC system connected to a QExcative Plus mass spectrometer. To create a spectral library that comprises peptides that correspond to the unmodified, lysylated and lysylated/hydroxylated form of EF-P initial data acquisition was performed in the data-dependent mode. The tryptic peptides of interest were included in a global inclusion list with their charge states *z*=2–5. Peptide identification was achieved by database searching with the MaxQuant software (version 1.5.2.8) (ref. 34) against the UniProt *E. coli* (K12) proteome. Lysylation (+128) and lysylation/hydroxylation (+144) were used as variable modifications. To build a spectral library, the results were further analysed using Skyline software (version 3.5) (ref. 35). Prominent charge states (unmodified peptide *z*=3, modified peptides *z*=4) were chosen for relative quantification by scheduled parallel reaction monitoring^36^. The resolution of tandem mass spectrometry scans was 35,000, the maximum ion time was 50 ms using an isolation window of 1 *m/z* and the collision energy of HCD fragmentation was 28 eV. The tryptic peptides of interest have a methionine close to the N terminus, oxidation of which leads to a +16 mass shift (identical to hydroxylation). As the degree of interfering oxidation may vary between different preparations, only *y*-ions that did not have this interference were used for quantification. The oxidation status was independent of the degree of modification. Parallel reaction monitoring transitions were extracted and integrated in Skyline at a resolution of 35,000. For quantification, the sum of peak areas of the seven most intense product ions was considered.

### Translation of model peptides

Translation was carried out in a cell-free coupled transcription–translation FIT system^23^. The reactions contained only five ARSs (MetRS, LysRS, GlyRS, AspRS and TyrRS) and five amino acids (Met, Lys, Gly, Asp and Tyr) together with *E. coli* total tRNA; under these conditions, free Pro is not charged onto the natural tRNA^Pro^ isoacceptors by ProRS. Transcription of the DNA templates into mRNAs was carried out by T7 RNA-polymerase present in the FIT system. Reactions were carried out at 37 °C for 20 min, except for the time-course analysis in 2.5 μl reaction mixture consisting of the following reagents: 50 mM HEPES-KOH (pH 7.6), 100 mM potassium acetate, 12.3 mM magnesium acetate, 2 mM ATP, 2 mM GTP, 1 mM CTP, 1 mM UTP, 20 mM creatine phosphate, 0.1 mM 10-formyl-5,6,7,8-tetrahydrofolic acid, 2 mM spermidine, 1 mM DTT, 1.5 mg ml^−1^ *E. coli* total tRNA, 1.2 μM *E. coli* ribosomes, 0.6 μM methionyl-tRNA formyltransferase, 2.7 μM IF1, 0.4 μM IF2, 1.5 μM IF3, 0.26 μM EF-G, 10 μM EF-Tu, 0.66 μM EF-Ts, 0.25 μM RF2, 0.17 μM RF3, 0.5 μM RRF, 4 μg ml^−1^ creatine kinase, 3 μg ml^−1^ myokinase, 0.1 μM inorganic pyrophosphatase, 0.1 μM nucleotide diphosphate kinase, 0.1 μM T7 RNA polymerase, 0.13 μM AspRS, 0.09 μM GlyRS, 0.11 μM LysRS, 0.03 μM MetRS, 0.02 μM TyrRS, 0.05 mM [^14^C]-aspartic acid, 0.5 mM glycine, 0.5 mM lysine, 0.5 mM methionine, 0.5 mM tyrosine and 50 μM aminoacyl-tRNA that is under investigation, where indicated, 3 μM lysylated EF-P and 0.5 μM DNA template (5′- GGCGT AATAC GACTC ACTAT AGGGT TAACT TTAAG AAGGA GAAAA ACATG AAGAA GAAGX XXXXX GGTGA CTACA AGGAC GACGA CGACA AGTAA GCTTC G -3′). For testing native tRNA^Pro^, 50 μM native or transcribed [^14^C]Pro-tRNA^Pro1/Pro2/Pro3^ mixture and 0.5 mM cold aspartic acid were added instead of cold Pro-tRNA and [^14^C]-aspartic acid. The reactions were stopped by addition of an equal volume of stop solution (0.9 M Tris-HCl (pH 8.45), 8% SDS, 30% glycerol and 0.001% xylene cyanol) and incubation at 95 °C for 2 min, and then analysed by 15% tricine SDS–PAGE and autoradiography. Intensity bands of interest were normalized to the total [^14^C]-aspartic acid intensity (125 pmol) included in the reaction mixture.

For translation of FLK-FLAG, 0.5 μM template DNA coding for FLK-FLAG (5′- GGCGT AATAC GACTC ACTAT AGGGT TAACT TTAAG AAGGA GAAAA ACATG ATACA ACCTA TTTCC GGCCC TCCTC CTGGG CAACC ACCAG GTCAG GGAGA TAATC TGGAC TACAA GGACG ACGAC GACAA GTAAG CTTCG -3′), 0.38 μM AsnRS, 0.06 μM GlnRS, 0.4 μM IleRS, 0.04 μM LeuRS, 0.04 μM SerRS and 0.5 mM each of asparagine, glutamine, isoleucine, leucine and serine were added to the above reaction mixture. Wild-type or C13G Pro-tRNA^Pro1/Pro2/Pro3^ mixture was used for Pro incorporation.

### Assay validation

To screen for EF-P interaction determinants in tRNA^Pro^, we established an assay using the FIT system described above. The reaction was performed in the absence or presence of EF-P. To measure the EF-P effect by point measurements we selected a strong stalling motif (Pro-Pro-Gly)^16^,^37^,^38^ (Supplementary Fig. 1b). When such strong stalling occurs on synthesis of short peptides, ribosomes release some of the peptidyl-tRNA. The extent of the peptidyl-tRNA drop-off is proportional to the duration of stalling. Therefore, the proline-induced stalling—and the EF-P effect—are not only reflected in the differences in the translation rates but also in the final product level, enabling us to assess the EF-P effect through quantifying the peptide yield of translation. To optimize the assay, we first measured the time courses of reactions and the optimum EF-P concentration (Supplementary Fig. 2a,b); in all subsequent experiments, the incubation time was set for 20 min and EF-P concentration 3 μM. Next, we validated the EF-P effect and established the dynamic range of the assay by using three different mRNA constructs (mR1, mR2 and mR3) coding for Pro-Gly-Gly, Pro-Pro-Gly and Pro-Pro-Pro, respectively (Supplementary Fig. 1). EF-P significantly improved translation yields of peptides with consecutive Pro residues (3.5- and 14.4-fold increase for P2 and P3, respectively), consistent with previous studies^11^,^12^,^16^. In contrast, the yield of the peptide P1 with a single Pro residue was significantly higher and not affected by the presence of EF-P, presumably due to a lack of stalling, establishing the dynamic range of our assay. As we use tRNA transcripts that lack all posttranscriptional modifications (Supplementary Fig. 4b), we compared the EF-P effect in our assay using native and transcribed tRNA^Pro^ (Supplementary Fig. 4c). The modifications did not affect the translation yield, in agreement with previous reports suggesting that the tRNA^Pro^ transcript is fully active with respect to aminoacylation, ternary complex formation and translation^22^.

### Preparation of initiation and posttranslocation complex

Initiation complexes were prepared by incubation of 1 μM *E. coli* ribosomes, 3 μM mRNA (5′- GGCAAGGAGGUAAAUAAUGCCGGGUUUCAUU -3′), 1.5 μM IF1, 1.5 μM IF2, 1.5 μM IF3, 1 mM GTP and 3 μM [^14^C]fMet-tRNA^fMet^ or f[^35^S]Met-tRNA^fMet^ in TAKM_7_ buffer (50 mM Tris-HCl (pH 7.5), 70 mM NH_4_Cl, 30 mM KCl and 7 mM MgCl_2_) for 30 min at 37 °C.

EF-Tu·GTP·aminoacyl-tRNA ternary complexes used to form posttranslocation complexes were prepared by incubating 32 μM EF-Tu, 0.1 mg ml^−1^ pyruvate kinase, 3 mM phosphoenolpyruvate and 1 mM GTP for 15 min at 37 °C in HiFi buffer (50 mM Tris-HCl (pH 7.5), 70 mM NH_4_Cl, 30 mM KCl, 3.5 mM MgCl_2_, 0.5 mM spermidine, 8 mM putrescine and 2 mM DTT), followed by addition of Pro-tRNA in a 1:2 ratio to EF-Tu. Ternary complexes used for quench-flow experiments were prepared by incubation of 100 μM EF-Tu, 0.1 mg ml^−1^ pyruvate kinase, 3 mM phosphoenolpyruvate and 1 mM GTP in HiFi buffer, and addition of [^3^H]Gly-tRNA^Gly3^ in a 1:2 ratio to EF-Tu. Posttranslocation complexes were prepared by mixing initiation complexes with a twofold excess of EF-Tu·GTP·Pro-tRNA and 0.1-fold of EF-G, and purified by size-exclusion chromatography (BioSuite 250, 5 μm HR SEC, Waters).

### fMet-Pro-Gly and fMet–Pmn formation

Synthesis of the tripeptide fMetProGly was initiated by mixing 0.1 μM purified posttranslocation complex (f[^14^C]MetPro-tRNA) and 2.5 μM ternary complex ([^3^H]Gly-tRNA^Gly3^) in HiFi buffer (pH 7.0, in the experiments with tRNA^Pro1^ variants) or TAKM_7_ buffer (pH 7.0, in the experiments with tRNA^Ser4^ variants) at 37 °C using a quench-flow apparatus (KinTek Laboratories, Inc.). Lysylated EF-P (3 μM) was present in both solutions. Reactions were quenched by addition of 0.5 M KOH, incubated for 30 min at 37 °C, followed by neutralization with acetic acid. Tripeptides were analysed by reverse-phase HPLC (Chromolith performance RP-8e 100-4.6 mm column, Millipore) using a 0–65% acetonitrile gradient in 0.1% trifluoroacetic acid and quantified by liquid scintillation counting. Rate constants of the peptide bond formation were calculated by exponential fitting using Prism 6 (GraphPad Software, Inc.), in which values for tRNA^Pro1^ variants and tRNA^Ser4^ variants were analysed by one-phase and two-phase fitting, respectively. For the values of tRNA^Ser4^ variants, weighted averages of two rate constants (*k*_fast_ and *k*_slow_) were calculated.

For fMet–Pmn formation, 0.2 μM initiation complex containing f[^35^S]Met-tRNA^fMet^ was incubated with 3 μM EF-P for 2 min and then mixed with an equal volume of 2 μM Pmn and incubated for further 5 min at 37 °C. The reaction was stopped by adding 1/10 volume of formic acid. fMet–Pmn was then mixed with 1.5 M sodium acetate saturated with MgSO_4_ at pH 4.5 and extracted by ethyl acetate according to the published protocol^39^, and quantified by liquid scintillation counting.

### Data availability

**Figure 1:**
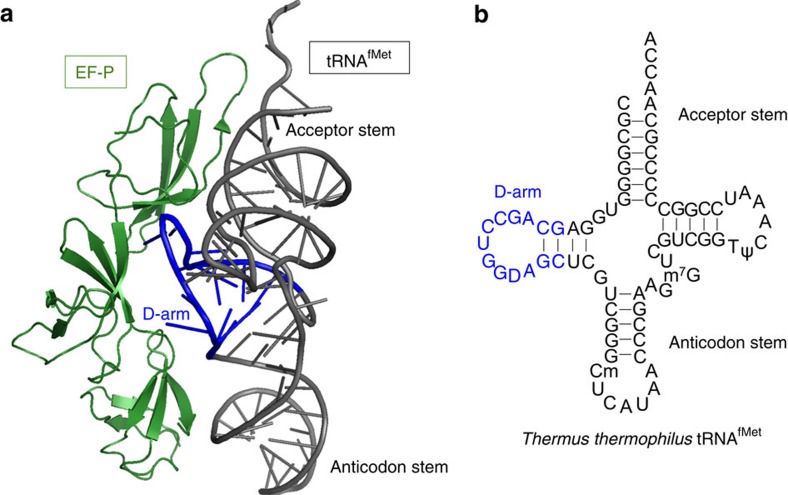
Structure of *T. thermophilus* EF-P and P-site tRNA^fMet^. (**a**) *T. thermophilus* EF-P and P-site tRNA^fMet^ bound to the 70S ribosome. The data were obtained from the Protein Data Bank (PDB) ID:4V6A. EF-P is shown in green and the tRNA in grey, except for the D-arm, which is shown in blue. The ribosome and mRNA are omitted from the figure for simplicity. (**b**) Secondary structure of *T. thermophilus* tRNA^fMet^. The D-arm region close to EF-P is indicated in blue. Cm, 2'-*O*-methylcytidine D, dihydrouridine; m^7^G, 7-methylguanosine; T, ribothymidine; ψ, pseudouridine.

**Figure 2:**
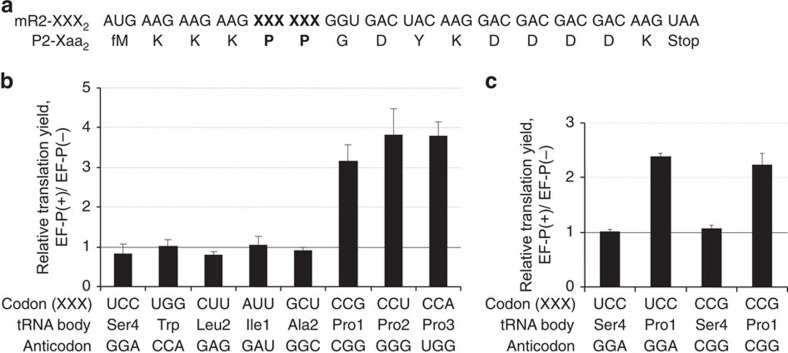
The sequence of tRNA^Pro^ is crucial for EF-P function. (**a**) mRNA sequence (mR2-XXX_2_) and the corresponding peptide sequence (P2-Xaa_2_) used in the experiment. (**b**) Relative translation yields of P2-Xaa_2_ peptides using different tRNA species as indicated. Codon (XXX) indicates the sequence of codons used for tandem Pro incorporation. *In vitro* transcripts of the respective *E. coli* tRNAs were aminoacylated with Pro by means of flexizyme technology and used for incorporation of Pro at the corresponding XXX codons. Values represent relative translation yields obtained with and without EF-P and calculated as [EF-P(+)/EF-P(−)]. Error bars, s.d. (*n*=3). See Supplementary Fig. 3a for the absolute peptide yield. (**c**) Effect of anticodon substitutions in tRNA^Ser4^ or tRNA^Pro1^. See Supplementary Fig. 3b for the absolute yield.

**Figure 3:**
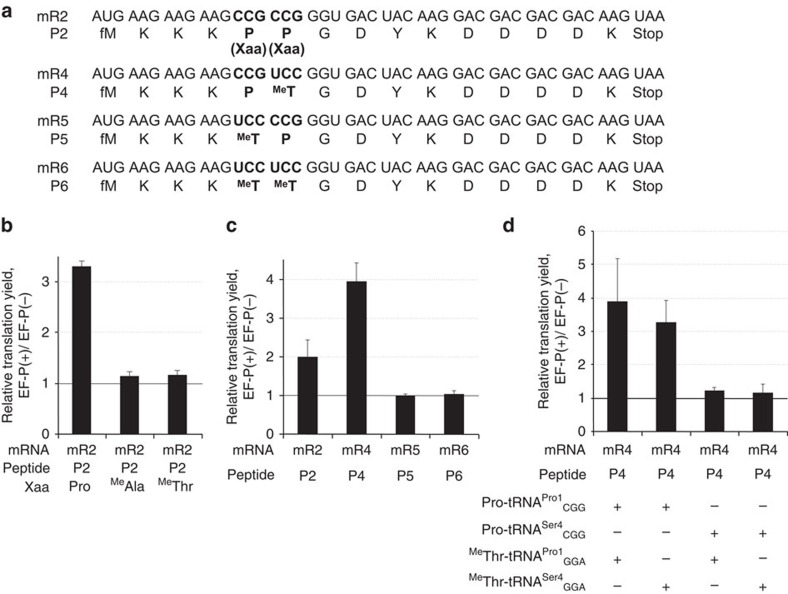
Effect of non-proteinogenic secondary amino acids. (**a**) Sequences of mRNAs (mR2, mR4-mR6) and their corresponding peptides (P2, P4-P6) used in the experiment. (**b**) Effect of EF-P on tandem incorporation of Pro or *N*-methyl-amino acids (Xaa). ^Me^Ala, *N*-methylalanine; ^Me^Thr, *N*-methylthreonine; Pro, proline. tRNA^Pro1^_CGG_ was used for incorporation of Xaa at CCG codons. See Supplementary Fig. 3c for the absolute peptide yield. (**c**) The importance of the first Pro in the sequence. Pro-tRNA^Pro1^_CGG_ and ^Me^Thr-tRNA^Pro1^_GGA_ were used for incorporation of Pro and ^Me^Thr at CCG and UCC codons, respectively. See Supplementary Fig. 3d for the absolute yield. (**d**) Dependence on tRNA sequence. Pro-tRNA^Pro1^_CGG_ and Pro-tRNA^Ser4^_CGG_ were used for incorporation of Pro at CCG codons, and ^Me^Thr-tRNA^Pro1^_GGA_ and ^Me^Thr-tRNA^Ser4^_GGA_ for incorporation of ^Me^Thr at UCC codons. See Supplementary Fig. 3e for the absolute yield. Error bars, s.d. (*n*=3).

**Figure 4:**
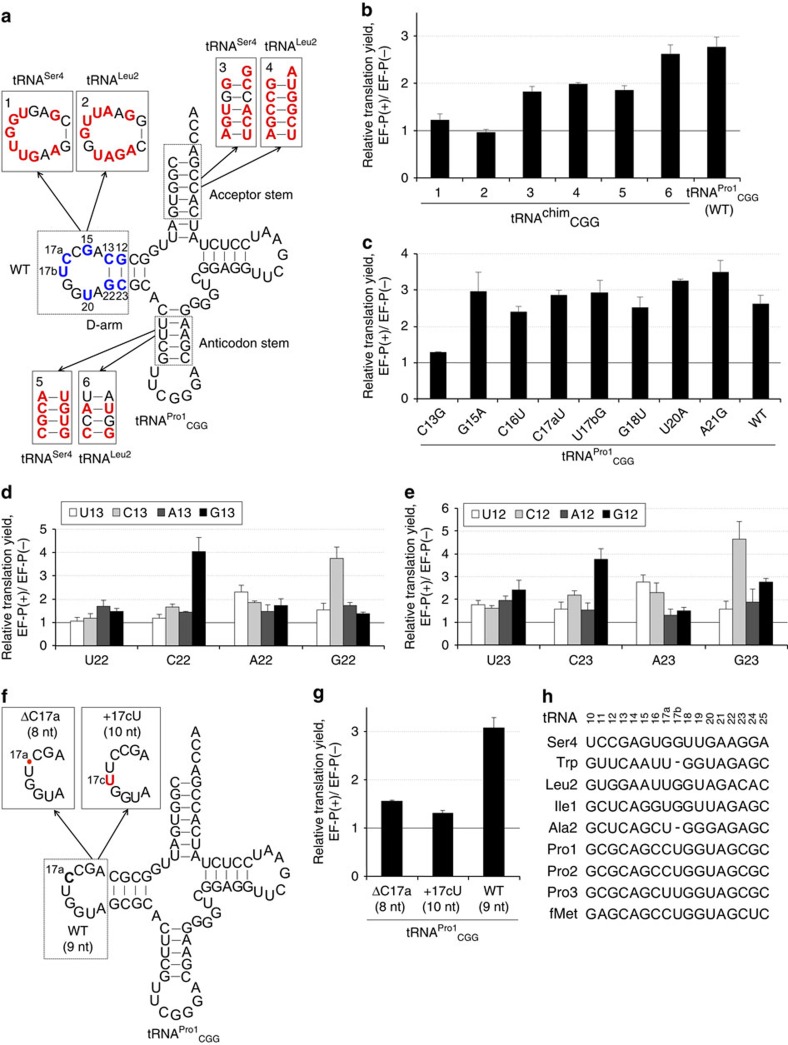
Identification of the tRNA^Pro1^ region required for EF-P function. mR2 (Fig. 3a) was used for translation. (**a**) Chimeric tRNA^Pro1^ with substitutions of the D arm, acceptor stem and anticodon stem using the corresponding sequences of tRNA^Ser4^ and tRNA^Leu2^. Bases that differ from the wild-type tRNA^Pro^ are highlighted in red or blue. (**b**) EF-P enhancement of translation with chimeric tRNAs or wild-type tRNA^Pro1^_CGG_. See Supplementary Fig. 3f for absolute peptide yield. (**c**) Effect of D-arm point mutations. See Supplementary Fig. 3g for absolute yield and also Supplementary Fig. 4a for results of multiple point mutants. D-stem point mutations of N13:N22 (**d**) and N12:N23 (**e**). See Supplementary Fig. 3h,i for absolute yield. (**f**) Schematics of tRNA^Pro1^ mutants with different D-loop lengths. (**g**) EF-P effect on translation with the D-loop tRNA^Pro1^ mutants as shown. See Supplementary Fig. 3j for absolute yield. (**h**) Comparison of the D-arm sequences of various *E. coli* tRNAs. Nucleotide modifications are not shown, because tRNA^Pro^ modifications are not required for EF-P activity.

**Figure 5:**
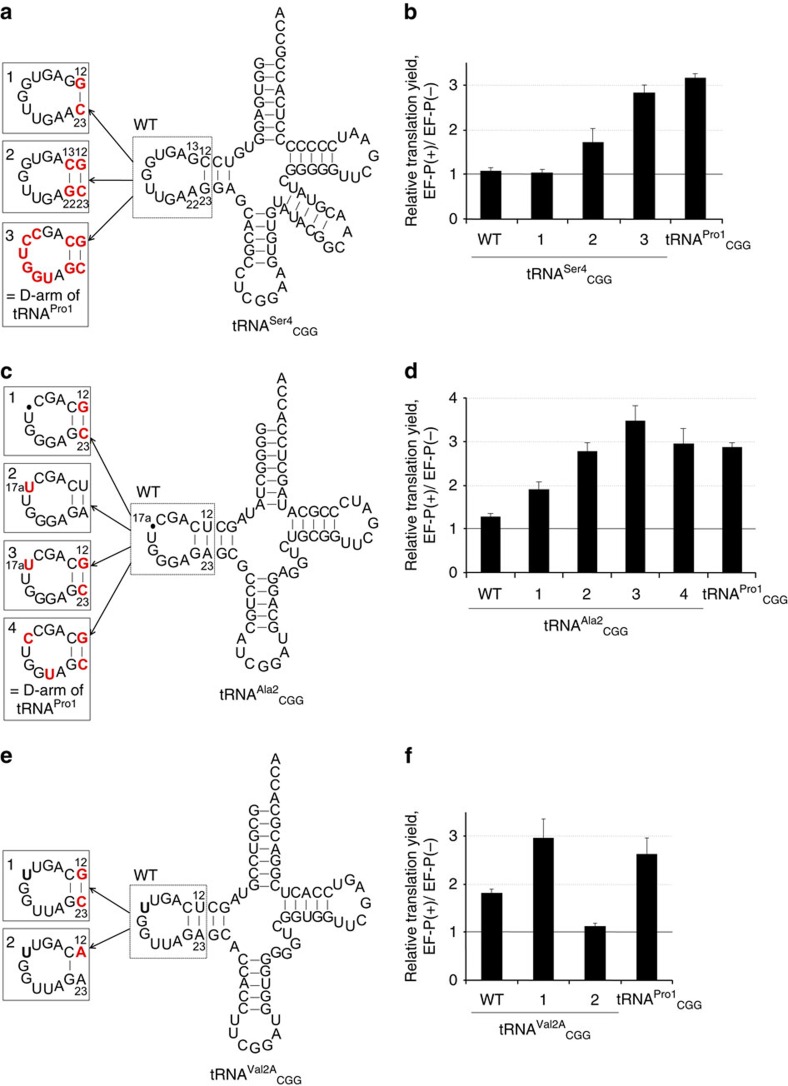
Implanting the tRNA^Pro1^ D-arm into inactive tRNAs. Structures of the D-arm mutants of tRNA^Ser4^ (**a**), tRNA^Ala2^ (**c**) and tRNA^Val2A^ (**e**). Bases that differ from the wild-type tRNAs are highlighted in red. (**b**,**d**,**f**) EF-P-mediated enhancement of mR2-CCG_2_ translation with the D-arm mutants. Error bars, s.d. (*n*=3). See Supplementary Fig. 3k–m for the absolute peptide yield.

**Figure 6:**
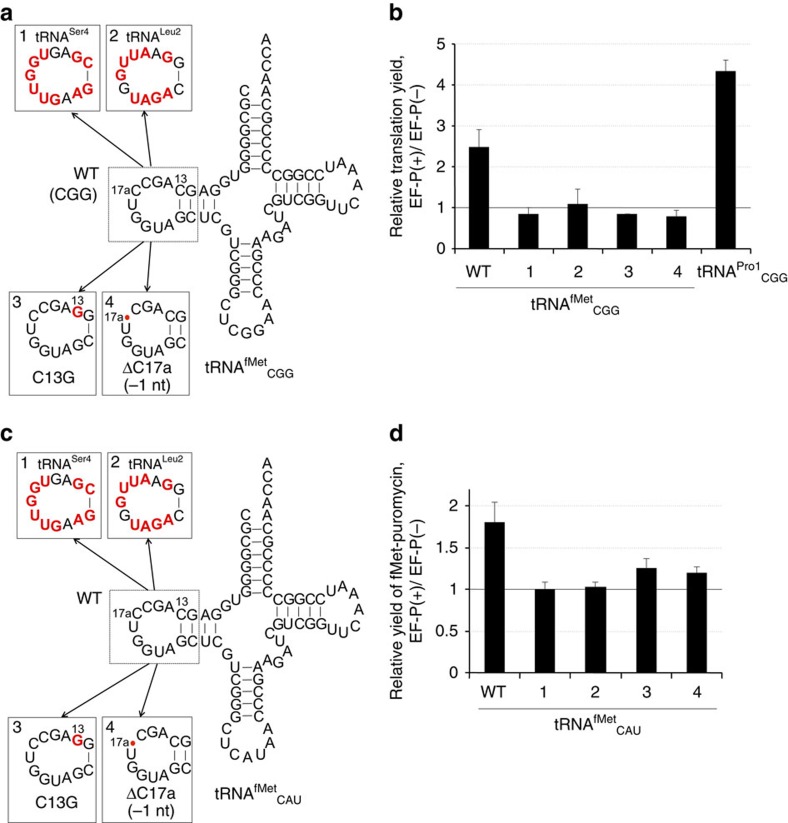
D-arm variants of tRNA^fMet^. (**a**) tRNA^fMet^_CGG_ variants bearing D-arm substitutions. Bases that differ from the wild-type tRNA^fMet^ are highlighted in red. (**b**) Translation of mR2 mRNA using the Pro-tRNA^fMet^_CGG_ variants. Relative translation yields of the P2 peptide are shown. See Supplementary Fig. 3n for absolute peptide yields. (**c**) tRNA^fMet^_CAU_ variants with D-arm substitutions. (**d**) fMet–Pmn formation using f[^35^S] Met-tRNA^fMet^_CAU_ variants. Error bars, s.d. (*n*=3). See Supplementary Fig. 3o for the yields of fMet–Pmn.

**Figure 7:**
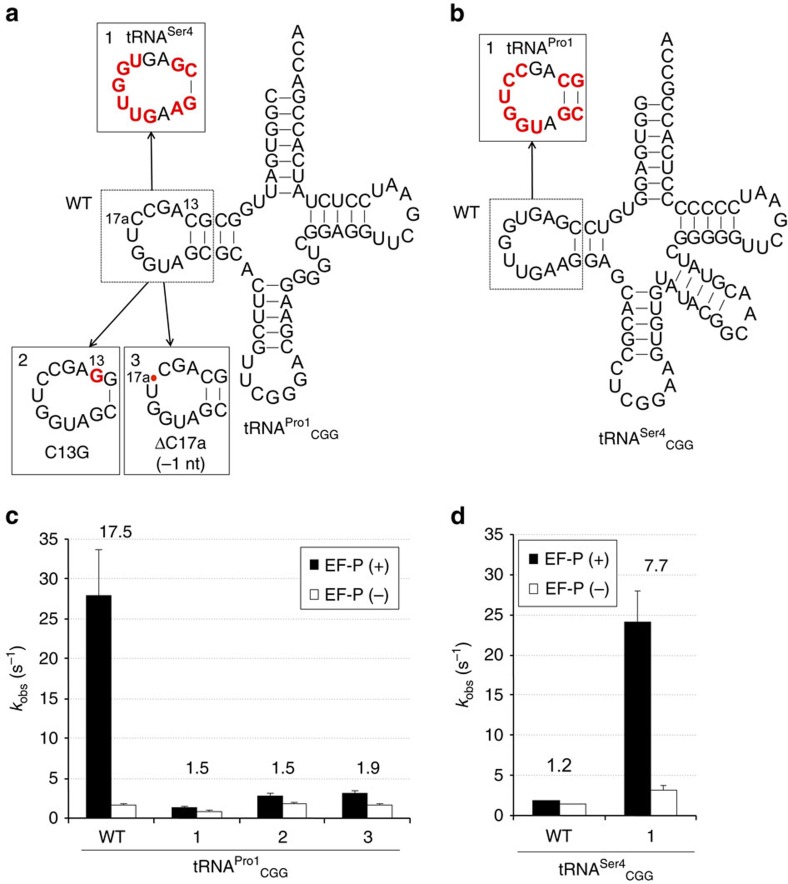
Kinetic effects of D-arm variants of tRNA^Pro1^ on peptide bond formation between Pro and Gly. Schematics of (**a**) the D-arm mutants of tRNA^Pro1^ and (**b**) the tRNA^Ser4^ variants used in these experiments. Bases that differ from the wild-type tRNAs are highlighted in red. (**c**,**d**) Rates of peptide bond formation between fMetPro and Gly (k_obs_) determined by exponential fitting of the time courses shown in Supplementary Fig. 6. Numbers above the bars indicate the ratio of *k*_obs_ calculated by [EF-P(+)/EF-P(−)]. Error bars, s.d.

The authors declare that all the data supporting the findings of this study are available within the article and its Supplementary Information files. Structural data of *T. thermophilus* EF-P and P-site tRNA^fMet^ bound to the 70S ribosome referenced in Fig. 1 are available in the RCSB Protein Data Bank (PDB) with ID 4V6A (http://www.rcsb.org/pdb/explore.do?structureId=4V6A)^40^.

Sequence data of tRNAs used in this study are available in GtRNAdb, http://gtrnadb.ucsc.edu/.

## Additional information

**How to cite this article:** Katoh, T. *et al*. Essential structural elements in tRNA^Pro^ for EF-P-mediated alleviation of translation stalling. *Nat. Commun.* 7:11657 doi: 10.1038/ncomms11657 (2016).

## Supplementary Material

Supplementary Information Supplementary Figures 1 - 7 and Supplementary Tables 1 and 2

Supplementary Table 3 List of tRNAs and primers used in this study. Gm indicates 2'-O-methylguanosine.
