# Supplementary material for: Essential structural elements in tRNAPro for EF-P-mediated alleviation of translation stalling
Source: Nat Commun. 2016 May 24;7:11657. doi: 10.1038/ncomms11657 (PMC4890201; doi:10.1038/ncomms11657)
Supplement: Supplementary Information — Supplementary Figures 1 - 7 and Supplementary Tables 1 and 2 [file ncomms11657-s1.pdf]

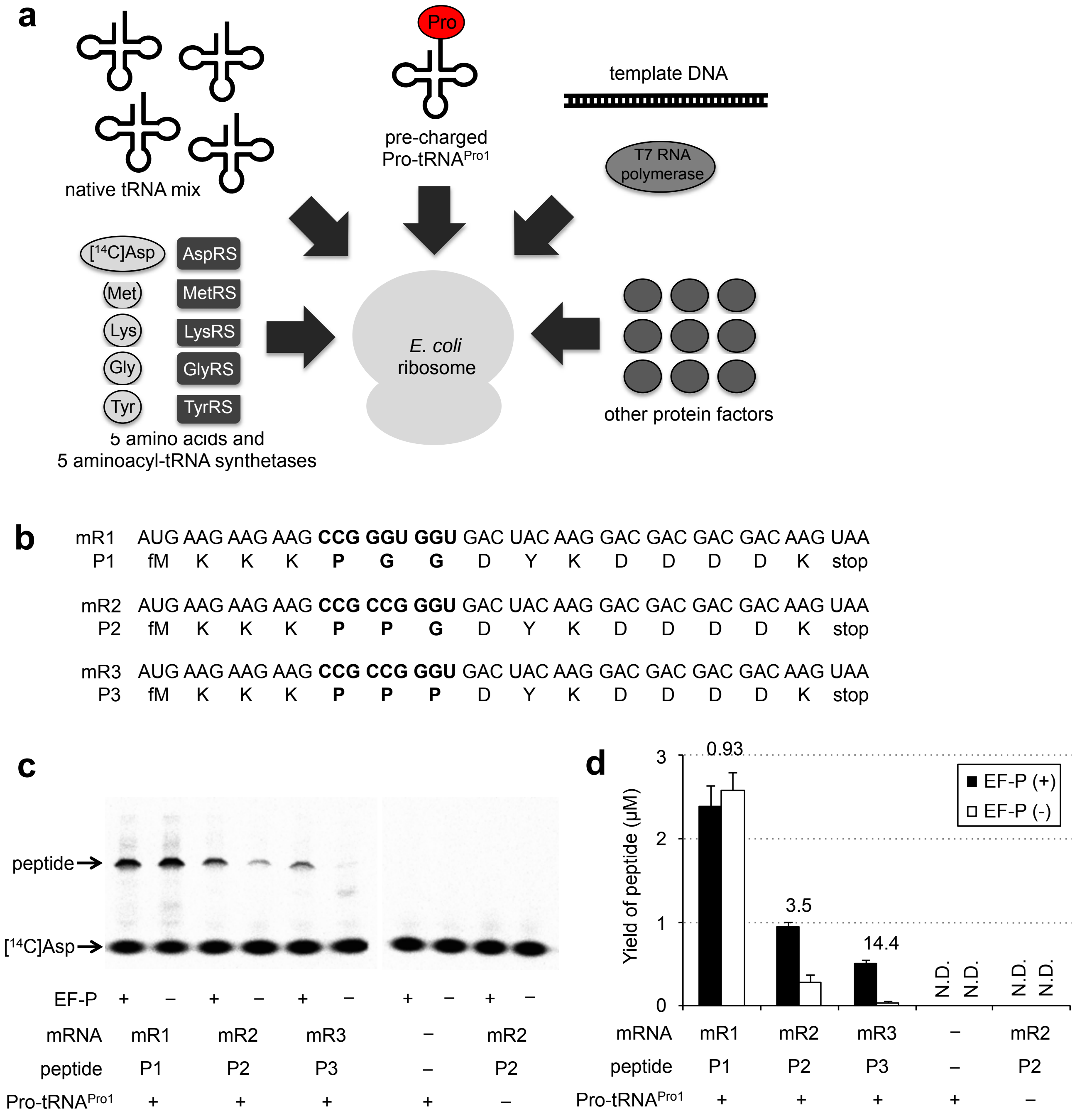

Supplementary Figure 1 EF-P effect on the synthesis of Pro-containing sequences in the FIT system. (**a**) Schematic depiction of peptide translation in the FIT system. For incorporation of Pro, *in vitro* transcribed *E. coli* tRNA^{Pro1} was pre-charged with Pro by means of the flexizyme technology and added to the translation system. Other amino acids were charged on natural *E. coli* tRNAs *de novo* by the corresponding aminoacyl-tRNA synthetases (ARSs) present in the FIT system. [^{14}C]Asp was used for autoradiographic detection of peptides. (**b**) Sequences of mRNAs (mR1–3) and the corresponding peptide sequences (P1–3). These mRNAs were transcribed from the corresponding DNA templates by T7 RNA polymerase present in the FIT system. (**c**) Tricine SDS-PAGE analysis of the peptides (P1-P3) synthesized in the FIT system. Translation was carried out for 20 min at 37°C in a 2.5-μL reaction mixture in the presence (3 μM) or absence of EF-P. (**d**) Yields of the full-length peptides (P1–3) synthesized by the FIT system. The [^{14}C]Asp-labeled peptide in the tricine SDS-PAGE gel was quantified by autoradiography. Numbers above the bars indicate the relative translation yields of the peptides calculated by [EF-P(+)/EF-P(–)]. N.D.: "not detected". Error bars, s.d. (n = 3).

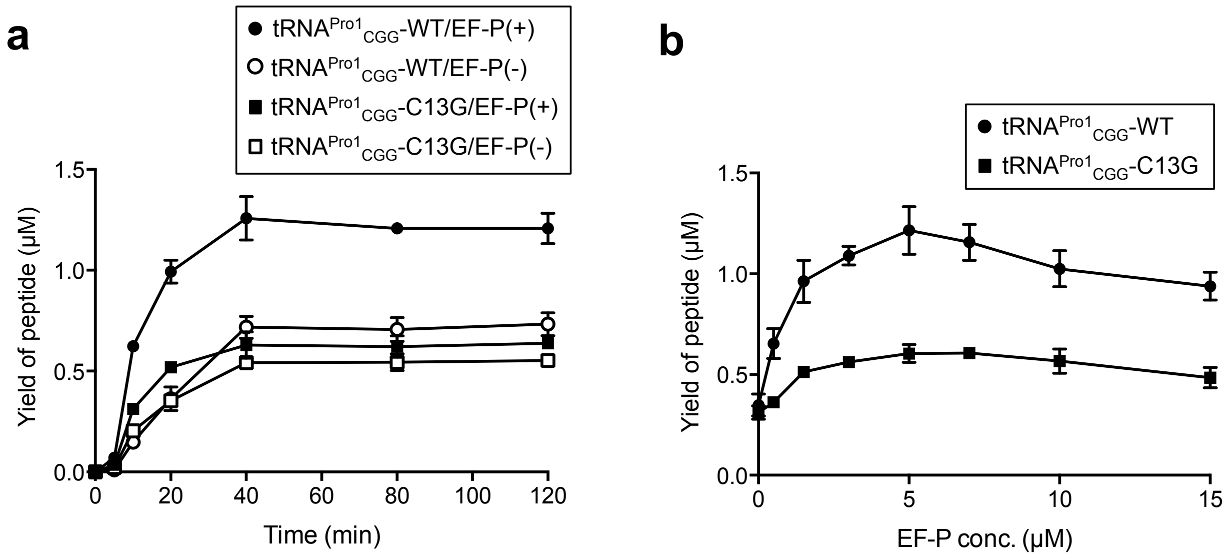

Supplementary Figure 2 Time-course analysis and titration of EF-P concentration in translation. (**a**) Time-course analysis in translation of P2-Pro_2 (Fig. 2a) using wildtype (WT) $tRNA^{Pro1}_{CGG}$ and C13G mutant in the presence (3 μM) and absence of EF-P. Error bars, s.d. (n = 3). (**b**) Titration of EF-P concentration in P2-Pro_2 (Fig. 2a) translation using the wildtype (WT) $tRNA^{Pro1}_{CGG}$ and C13G mutant for Pro incorporation. Translation time is 20 min. Error bars, s.d. (n = 3).

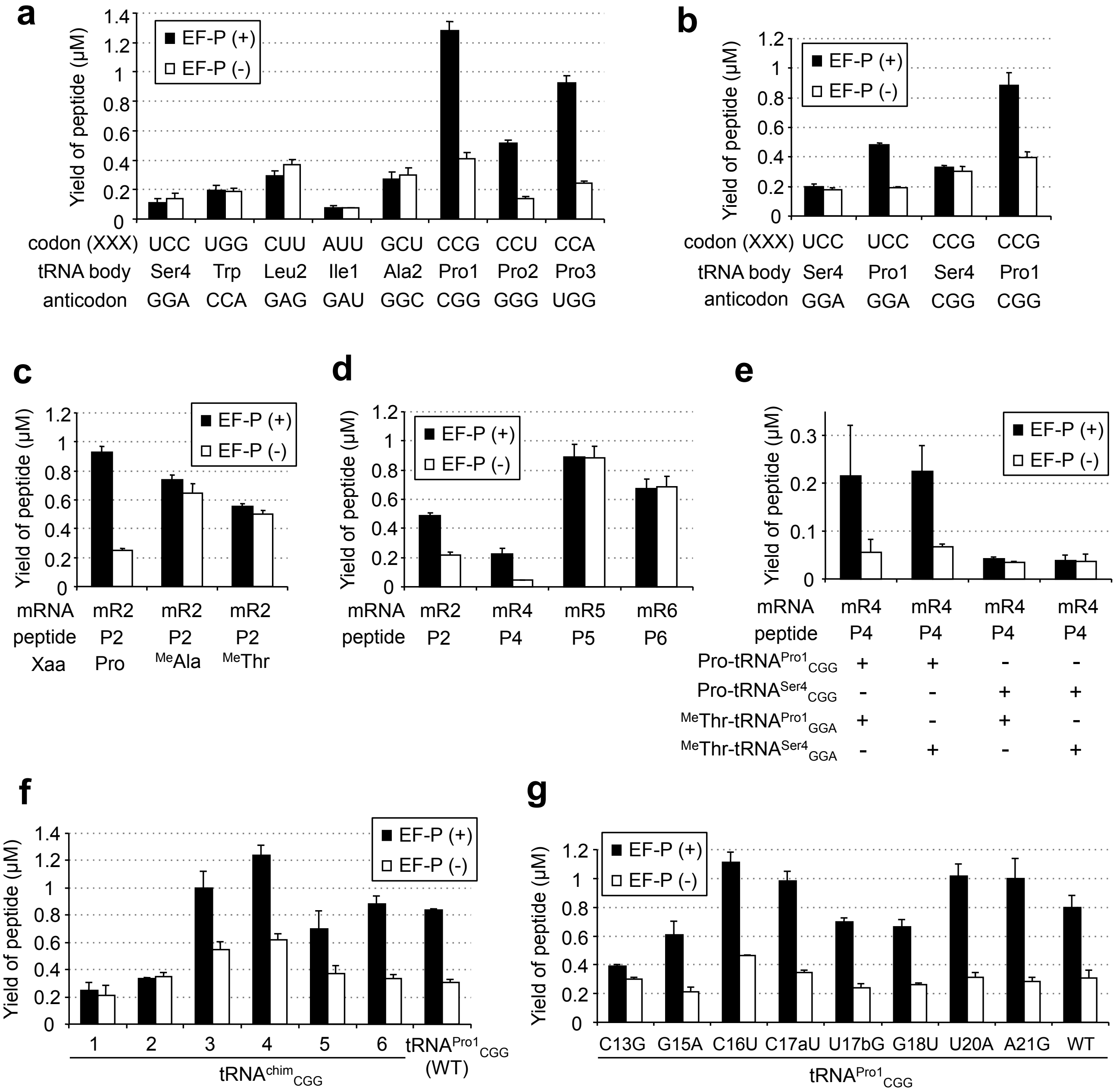

Supplementary Figure 3 Quantification of absolute translation yields of peptides. (**a-n**) Absolute translation yields of the peptides obtained in the experiments of Fig. 2b (**a**), Fig. 2c (**b**), Fig. 3b (**c**), Fig. 3d (**d**), Fig. 3e (**e**), Fig.4b (**f**), Fig. 4c (**g**), Fig. 4d (**h**), Fig.4e (**i**), Fig. 4g (**j**), Fig. 5b (**k**), Fig. 5d (**l**), Fig. 5f (**m**) and Fig. 6b (**n**). Translation products of each experiment were separated by 15% tricine SDS-PAGE, and the corresponding peptide labeled with [^{14}C]Asp were quantified by autoradiography. Black and white bars indicate results of translation with and without EF-P, respectively. Error bars, s.d. (n = 3). (**o**) Yields of f[^{35}S]Met-puromycin obtained in the experiment of Fig. 6d were analyzed by liquid scintillation counting. Black and white bars indicate results of reaction with and without EF-P, respectively. Error bars, s.d. (n = 3).

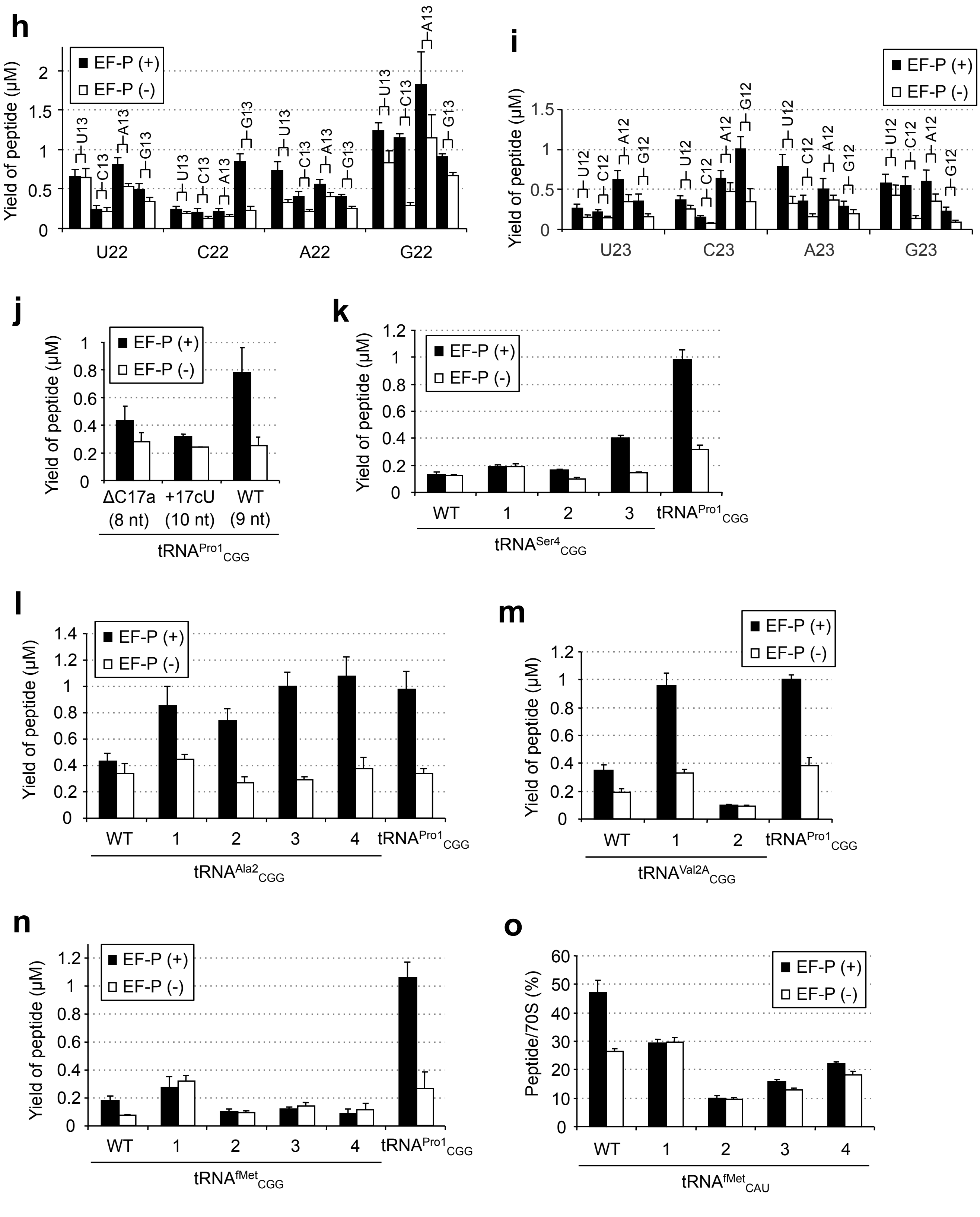

Supplementary Figure 3 (continued)

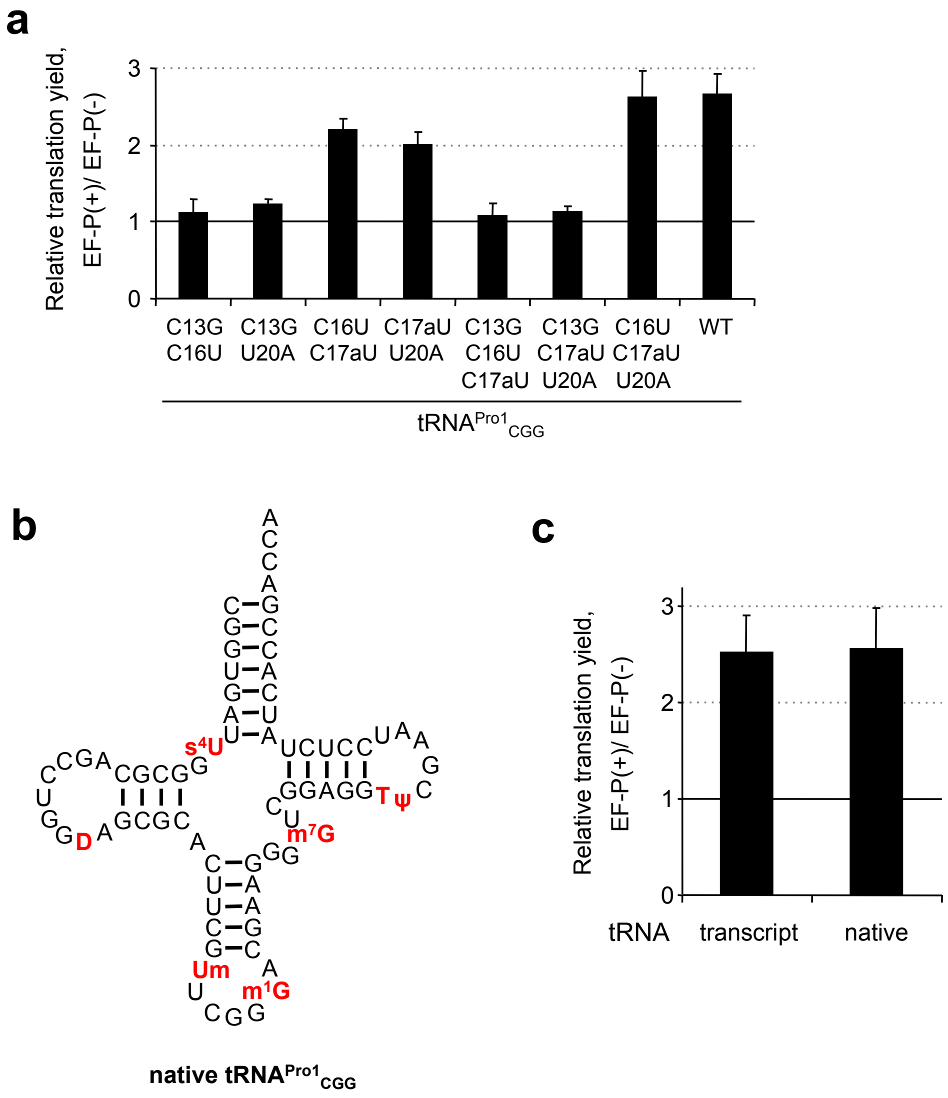

Supplementary Figure 4 Effects of multiple point mutations and nucleotide modifications. (**a**) Effect of double or triple point mutations introduced into $tRNA^{Pro1}_{CGG}$ on incorporation of Pro. Relative translation yields were analyzed using mR2-CCG_2 mRNA (Fig. 2a). Error bars, s.d. (n = 3). (**b**) Secondary structure of native $tRNA^{Pro1}_{CGG}$ with nucleotide modifications indicated by red. s^4U: 4-thiouridine, D: dihydrouridine, Um: 2'-*O*-methyluridine, m^1G: 1-methylguanosine, m^7G: 7-methylguanosine, T: ribothymidine, ψ: pseudouridine (**c**) Incorporation of [^{14}C]Pro at tandem CCG codons of mR2-CCG_2 mRNA using transcript and native $tRNA^{Pro1/Pro2/Pro3}$ mixture. Values represent relative translation yields calculated as [EF-P(+)/EF-P(–)]. Error bars, s.d. (n = 3).

a

mRNA	AUG	AUA	CAA	**CCU**	AUU	UCC	GGC	**CCU**	**CCU**	**CCU**	GGG	CAA	**CCA**	**CCA**	GGU
FLK-FLAG	fM	I	Q	**P**	I	S	G	**P**	**P**	**P**	G	Q	**P**	**P**	G

CAG	GGA	GAU	AAU	CUG	GAC	UAC	AAG	GAC	GAC	GAC	GAC	AAG	UAA
Q	G	D	N	L	D	Y	K	D	D	D	D	K	stop

b

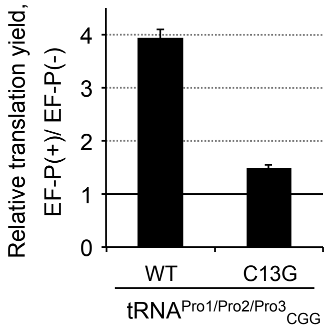

Supplementary Figure 5 Improvement of translation of FLK-FLAG by EF-P depending on D-arm structure of tRNA^{Pro} used for Pro incorporation. (**a**) Sequence of mRNA and FLK-FLAG peptide, 20 amino acids from the N terminus of FLK protein followed by FLAG sequence. (**b**) Improvement of translation yield of FLK-FLAG by EF-P. Pro was pre-charged on wildtype tRNA$^{Pro1/Pro2/Pro3}$ mixture or their D-arm mutants (C13G), and used for Pro incorporation in translation of FLK-FLAG. Translation product was separated by 15% tricine SDS-PAGE, and the desired peptide labeled with [^{14}C]Asp was quantified by autoradiography. Values represent relative translation yields calculated as [EF-P(+)/EF-P(–)]. Error bars, s.d. (n = 3).

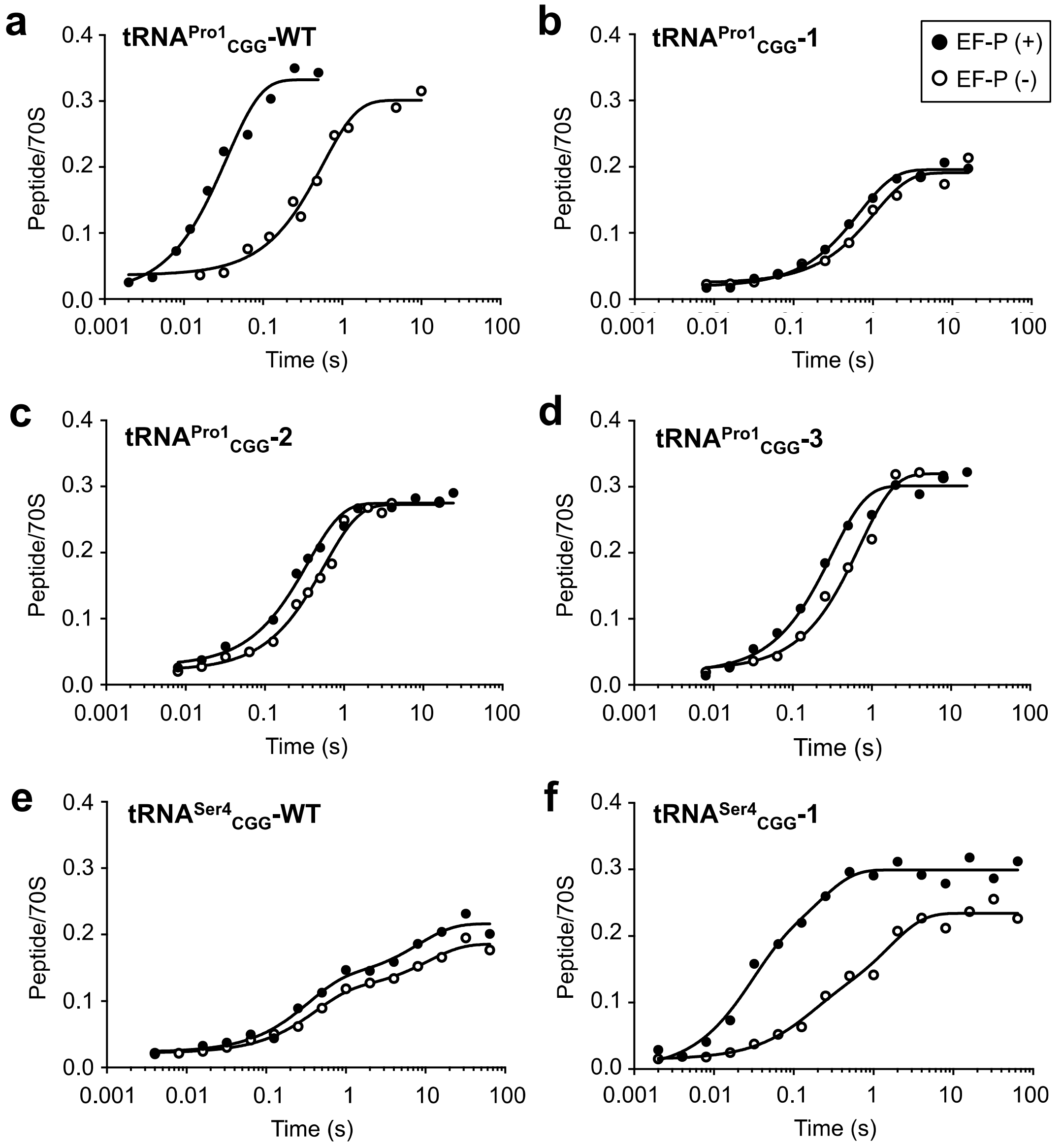

Supplementary Figure 6 Time courses of peptide bond formation between fMet-Pro and Gly using D-arm variants of tRNA^{Pro1} and tRNA^{Ser4}. The reaction was initiated by mixing posttranslocation complex containing [^{14}C]fMet-Pro-tRNA and [^{3}H]Gly-tRNA^{Gly3}•EF-Tu•GTP ternary complex, and reaction products were analyzed by HPLC. Posttranslocation complexes were prepared with the 6 different Pro-tRNA variants shown in Fig. 6a,b.

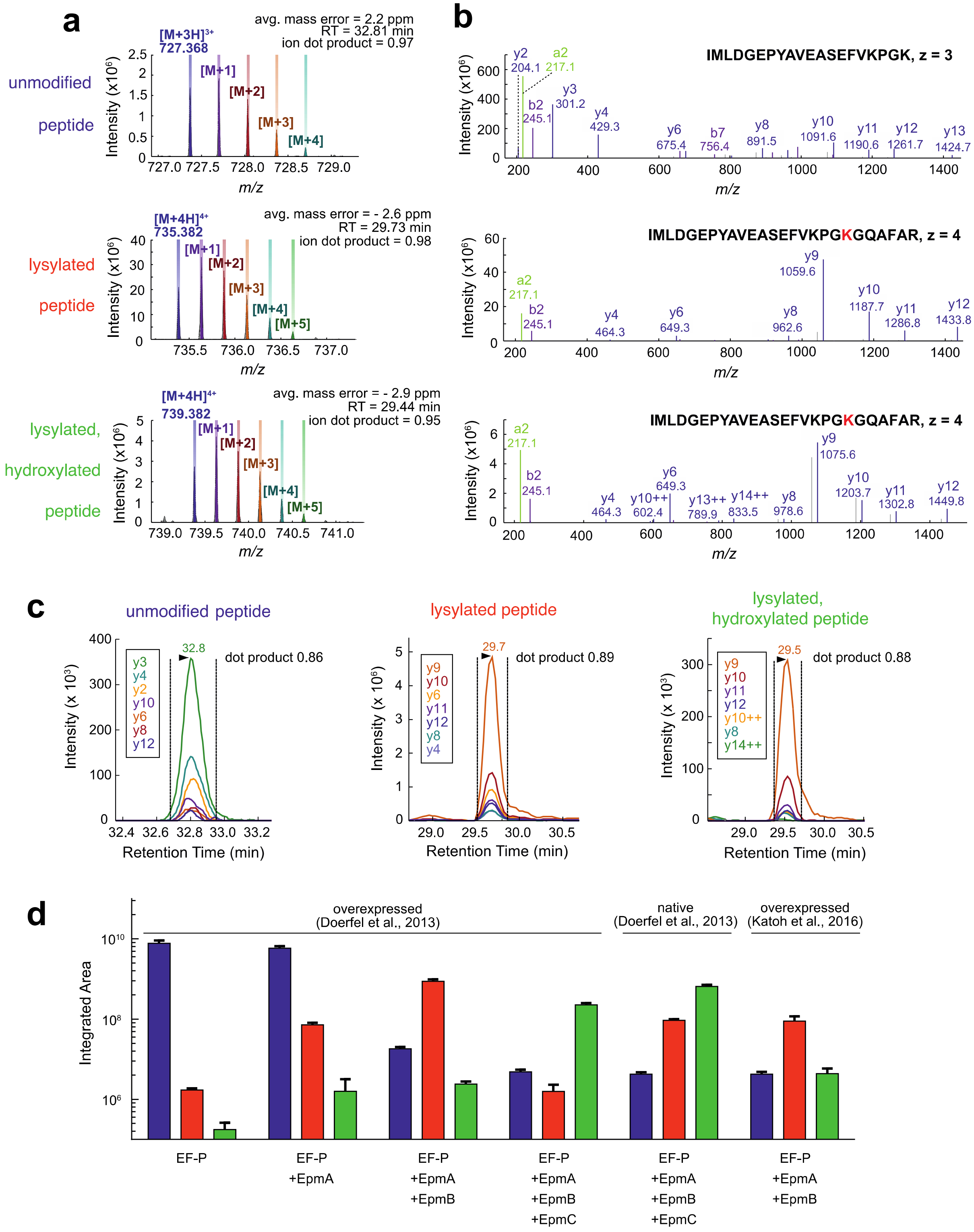

Supplementary Figure 7 Mass spectrometric analysis of EF-P modification. (**a**) Mass spectra of the unmodified (z=3; m/z=727.3696 (monoisotopic ion)) and modified peptides (z=4; lysylated m/z=735.3837; lysylated/hydroxylated m/z=739.3825 (monoisotopic ions)). Series of other isotopic ions are indicated as [M+1~5]. (**b**) Library MS/MS spectra of the unmodified (z=3) and modified peptides (z=4). The modified lysine residue is indicated in red in the peptide sequence. (**c**) Elution profile of parallel reaction monitoring transitions (PRM). Peptides were identified by co-elution of their PRM transitions and comparison to the reference library spectrum (dot product). Example PRM profiles show the modification status of EF-P used in this work. (**d**) Relative label-free quantification of the EF-P modification status by PRM. Bars represent the sum of 7 PRM transitions. Error bars indicate standard deviations of three independent technical replicates.

	Sequence of D-arm	Loop (nt)	Stem (bp)	12,13/22,23
Ala1B	GCUCAGCUGGGAGAGC	8	4	UC/GA
Ala2	GCUCAGCUGGGAGAGC	8	4	UC/GA
Arg2	GCUCAGCUGGAUAGAGU	9	4	UC/GA
Arg3	GCUCAGCUGGAUAGAGC	9	4	UC/GA
Arg4	GCUCAGUUGGAUAGAGC	9	4	UC/GA
Arg5	GUUAAAUGGAUAUAAC	8	4	UA/UA
Asn	GUUCAGUCGGUAGAAC	8	4	UC/GA
Asp	GUUCAGUCGGUUAGAAU	9	4	UC/GA
Cys	ACAAAGCGGUUAUGU	9	3	AA/AU
Gln1	GCCAAGCGGUAAGGC	9	3	CA/AG
Gln2	GCCAAGCGGUAAGGC	9	3	CA/AG
Glu	GUCUAGAGGCCCAGGAC	9	4	CU/GG
Gly1	GUUCAAUGGUAGAAC	7	4	UC/GA
Gly2	GUAUAAUGGCUAUUAC	10	3	AU/UU
Gly3	GCUCAGUUGGUAGAGC	8	4	UC/GA
His	GCUCAGUUGGUAGAGC	8	4	UC/GA
Ile1	GCUCAGGUGGUUAGAGC	9	4	UC/GA
Ile2	GCUCAGUGGUUAGAGC	8	4	UC/GA
Leu1	GCGGAAUUGGUAGACGC	11	3	GG/AC
Leu2	GUGGAAUUGGUAGACAC	11	3	GG/AC
Leu3	GCGAAAUUGGUAGACGC	11	3	GA/AC
Leu4	GUGGAAUCGGUAGACAC	11	3	GG/AC
Leu5	GCGAAAUCGGUAGACGC	11	3	GA/AC
Lys	GCUCAGUUGGUAGAGC	8	4	UC/GA
Met	GCUCAGUUGGUUAGAGC	9	4	UC/GA
fMet	GAGCAGCCUGGUAGCUC	9	4	GC/GC
Phe	GCUCAGUCGGUAGAGC	8	4	UC/GA
Pro1	GCGCAGCCUGGUAGCGC	9	4	GC/GC
Pro2	GCGCAGCCUGGUAGCGC	9	4	GC/GC
Pro3	GCGCAGCUUGGUAGCGC	9	4	GC/GC
Sec	UCGUCUCCGGUGAGGCGG	4	7	GU/GC
Ser1	GCCGAGCGGUUGAAGGC	11	3	CG/AG
Ser2	CCGGAGCGGCUGAACGG	11	3	GG/AC
Ser3	GCCGAGAGGCUGAAGGC	11	3	CG/AG
Ser4	UCCGAGUGGUUGAAGGA	11	3	CG/AG
Ser5	UCCGAGUGGCUGAAGGA	11	3	CG/AG
Thr1	GCUCAGUUGGUAGAGC	8	4	UC/GA
Thr2	GCUCAGUUGGUAGAGC	8	4	UC/GA
Thr3	GCUCAGUUGGUAGAGC	8	4	UC/GA
Thr4	GCUCAGUAGGUAGAGC	8	4	UC/GA
Trp	GUUCAAUUGGUAGAGC	8	4	UC/GA
Tyr1	CCCGAGCGGCCAAAGGG	11	3	CG/AG
Tyr2	CCCGAGCGGCCAAAGGG	11	3	CG/AG
Val1	GCUCAGCUGGGAGAGC	8	4	UC/GA
Val2A	GCUCAGUUGGUUAGAGC	9	4	UC/GA
Val2B	GCUCAGUUGGUUAGAGC	9	4	UC/GA

Supplementary Table 1 Comparison of the D-arm sequences of *E. coli* tRNAs. Watson-Crick base-pairing (A/U and G/C) and wobble base-pairing (G/U) nucleotides are shown in red and blue, respectively. G/U wobble base pairs are also considered as base pairs for counting the length of the D-loop and stem region.

Prokaryote	Sequence of D-arm	Loop (nt)	Stem (bp)	12,13/22,23
Agrobacterium tumefaciens (CGG)	GCGCAGUCUGGUAGCGC	9	4	GC/GC
Bacillus subtilis (UGG)	GCUCAGCUUGGUAGAGC	9	4	UC/GA
Escherichia coli (CGG, Pro1)	GCGCAGCCUGGUAGCGC	9	4	GC/GC
Escherichia coli (UGG, Pro3)	GCGCAGCUUGGUAGCGC	9	4	GC/GC
Mycobacterium tuberculosis (CGG)	GCGCAGCUUGGUAGCGC	9	4	GC/GC
Mycoplasma pneumoniae (UGG)	GCUUAGUUUGGUAGAGC	9	4	UU/GA
Prochlorococcus marinus (UGG)	GCGCAGCUUGGUAGCGC	9	4	GC/GC
Pseudomonas aeruginosa (CGG)	GCGCAGCUUGGUAGCGC	9	4	GC/GC
Salmonella typhi (CGG)	GCGCAGCCUGGUAGCGC	9	4	GC/GC
Streptococcus pneumoniae (UGG)	GCUCAGCUUGGUAGAGU	9	4	UC/GA
Thermus thermophilus (CGG)	GCGCAGCCCGGUAGCGC	9	4	GC/GC

Eukaryote	Sequence of D-arm	Loop (nt)	Stem (bp)	12,13/22,23
Arabidopsis thaliana (CGG)	GUCUAGUGGUAUGAU	9	3	CU/UG
Caenorhabditis elegans (CGG)	GUCUAGAGGUAUGAU	9	3	CU/UG
Drosophila melanogaster (CGG)	GUCUAGAGGUAUGAU	9	3	CU/UG
Homo sapiens (UGG)	GUCUAGGGGUAUGAU	9	3	CU/UG
Homo sapiens (CGG)	GUCUAGGGGUAUGAU	9	3	CU/UG
Mus musculus (CGG)	GUCUAGGGGUAUGAU	9	3	CU/UG
Plasmodium falciparum (UGG)	GUCUAGUGGUAUGAU	9	3	CU/UG
Saccharomyces cerevisiae (UGG)	GUCUAGUGGUAUGAU	9	3	CU/UG
Zea mays (CGG)	GUCUAGUGGUAUGAU	9	3	CU/UG

Archaea	Sequence of D-arm	Loop (nt)	Stem (bp)	12,13/22,23
Halobacterium salinarum (CGG)	GGGUAGCUUGGUAUCC	6	5	GU/AU
Haloferax volcanii (CGG)	GGGUAGCUUGGUAUCC	6	5	GU/AU
Methanococcus jannaschii (UGG)	GGGUAGCCUGGUCUAUCC	6	6	GU/AU
Pyrobaculum aerophilum (CGG)	GUCUAGCCUGGUAGGAU	9	4	CU/GG
Nitrosopumilus maritimus (UGG)	GUCUAGCUUGGUAUGAU	11	3	CU/UG
Pyrococcus abyssi (CGG)	GGGUAGCUUGGCCUAUCC	6	6	GU/AU
Sulfolobus islandicus (CGG)	GUCUAGCCUGGACUAGGAU	11	4	CU/GG
Thermoplasma volcanium (CGG)	GGGUAGCCUGGUAUCCU	11	3	GU/UC
Thermococcus gammatolerans (CGG)	GGGUAGCUUGGUCUAUCCU	13	3	GU/UC

Supplementary Table 2 Comparison of the D-arm sequences among different organisms. Watson-Crick base-pairing (A/U and G/C) and wobble base-pairing (G/U) nucleotides are shown in red and blue, respectively. G/U wobble base pairs are also considered as base pairs for counting the length of the D-loop and stem.
